# ERβ/circAHNAK Axis Inhibits USP10–FMR1 Deubiquitination to Prevent m⁶A‐Mediated ADAM17 Decay and Promote Angiogenesis in Clear Cell Renal Cell Carcinoma

**DOI:** 10.1002/advs.202509654

**Published:** 2025-10-07

**Authors:** Chao Xu, Sujing Zhang, Yuepeng Liu, Jingwei Lv, Yilong Cao, Yao Chen, Hao Sun, Bowei Zhang, Shengtao Dai, Jiehan Li, Xiaoling Li, Bei Shi, Qingyun Sun, Meng Zhu, Junfei Gu

**Affiliations:** ^1^ Department of Urology The Second Hospital of Hebei Medical University 215 Heping West Road Shijiazhuang 050000 China; ^2^ Department of Nuclear Medicine The Second Hospital of Hebei Medical University 215 Heping West Road Shijiazhuang 050000 China; ^3^ Hebei Medical University 361 Zhongshan East Road, Shijiazhuang Hebei 050017 China

**Keywords:** ADAM17, angiogenesis, circAHNAK, clear cell renal cell carcinoma, deubiquitination, ERβ, m^6^A modification

## Abstract

Estrogen receptor β (ERβ), a nuclear hormone receptor, plays multifaceted roles in tumorigenesis, including transcriptional regulation and noncoding RNA signaling. In clear cell renal cell carcinoma (ccRCC), ERβ is frequently overexpressed and associated with enhanced angiogenesis, a hallmark of aggressive tumor progression. However, the mechanisms linking ERβ signaling to the post‐transcriptional and post‐translational regulation of angiogenic drivers remain poorly defined. In this study, circAHNAK is identified as a direct downstream effector of ERβ and is significantly upregulated in ccRCC tissues. Elevated circAHNAK in ccRCC cells promotes angiogenesis‐related phenotypes in HUVECs by stabilizing ADAM17, a metalloprotease known to facilitate tumor angiogenesis. Mechanistically, ERβ enhances circAHNAK expression by transcriptionally activating its host gene. CircAHNAK binds to FMR1, preventing USP10‐mediated deubiquitination of FMR1 at Lys593 and thereby accelerating FMR1 proteasomal degradation. In turn, loss of FMR1 impairs the recognition of m⁶A‐modified transcripts, thereby blocking METTL14‐mediated m⁶A‐dependent decay of ADAM17 mRNA. Consequently, ADAM17 accumulates in ccRCC cell‐derived exosomes, driving angiogenesis in ccRCC. Overall, these findings define a novel ERβ/circAHNAK/FMR1/ADAM17 axis that integrates USP10‐mediated deubiquitination with FMR1‐mediated m⁶A modification to promote ADAM17 expression and angiogenesis in ccRCC. Targeting this pathway may represent a promising therapeutic strategy for ccRCC.

## Graphical abstract

In clear cell renal cell carcinoma, estrogen receptor β (ERβ) upregulates circAHNAK, which competitively binds USP10 to prevent FMR1 deubiquitination, leading to FMR1 degradation. Loss of FMR1 hinders m⁶A‐dependent decay of ADAM17 mRNA, resulting in ADAM17 accumulation in tumor exosomes and enhanced angiogenesis. This ERβ/circAHNAK/FMR1/ADAM17 axis offers a new therapeutic target.

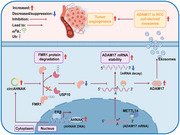

## 1 Introduction

Renal cell carcinoma (RCC) is among the most common malignancies of the genitourinary system, with clear cell RCC (ccRCC) accounting for over 80% of cases.^[^ ^1^, ^2^ ^]^ A hallmark of ccRCC is its highly vascular nature, largely driven by dysregulated hypoxia‐inducible factor (HIF) signaling and overexpression of vascular endothelial growth factor (VEGF). Although anti‐angiogenic therapies targeting VEGF or its receptors have improved outcomes in advanced ccRCC, intrinsic and acquired resistance, as well as limited therapeutic efficacy, remain major clinical challenges.^[^ ^3^, ^4^, ^5^ ^]^ Emerging evidence suggests that post‐transcriptional and post‐translational regulatory mechanisms may contribute to treatment evasion, but the precise molecular underpinnings remain unclear.^[^ ^6^, ^7^, ^8^ ^]^ Thus, elucidating novel pathways governing angiogenesis in ccRCC may provide the foundation for more effective therapeutic strategies.

Estrogen receptor beta (ERβ), a member of the nuclear receptor superfamily, is frequently upregulated in ccRCC and has been implicated in promoting tumor progression and angiogenesis.^[^ ^9^, ^10^ ^]^ Importantly, angiogenesis in ccRCC is orchestrated not only by transcriptional programs but also by diverse post‐transcriptional and post‐translational layers of regulation.^[^ ^8^, ^11^ ^]^ Hence, determining whether ERβ regulates pro‐angiogenic factors via post‐transcriptional and post‐translational routes could identify novel targets for ccRCC therapy.

Circular RNAs (circRNAs) are a class of non‐coding RNAs produced by back‐splicing of precursor mRNAs, increasingly recognized as key tumor regulators.^[^ ^12^ ^]^ Beyond functioning as competing endogenous RNAs (ceRNAs) that sequester microRNAs, circRNAs can modulate gene expression through interactions with RNA‐binding proteins (RBPs).^[^ ^13^ ^]^ For example, circNEIL3 promotes glioma progression by stabilizing IGF2BP3, while circPAK2 facilitates vasculogenesis and invasion in gastric cancer through the IGF2BPs/VEGFA signaling axis.^[^ ^14^, ^15^ ^]^ These findings underscore the critical roles of circRNA–RBP interactions in regulating tumor progression.

Ubiquitination is one of the most prevalent post‐translational modifications, modulating protein stability, localization, and activity by covalently attaching ubiquitin chains to target substrates.^[^ ^16^, ^17^ ^]^ Deubiquitination, the reversal of ubiquitination, is mediated by deubiquitinases (DUBs), which cleave ubiquitin moieties to regulate substrate turnover and function.^[^ ^18^ ^]^ USP10, a cysteine protease DUB, has emerged as a key player in cancer biology by stabilizing proteins such as p53 and CCND1, thereby modulating cell cycle progression, apoptosis, and DNA damage response.^[^ ^19^, ^20^ ^]^ Accordingly, USP10 is considered a promising target for therapeutic therapy.

N6‐methyladenosine (m⁶A), the most prevalent internal modification in eukaryotic mRNAs and non‐coding RNAs, occurs at consensus RRACH motifs (R = A/G; H = A/C/U).^[^ ^21^, ^22^ ^]^ m⁶A marks are recognized by a range of reader proteins, including but not limited to YTHDFs, HNRNPs, and IGF2BPs, which coordinate diverse aspects of RNA metabolism such as splicing, translation, and degradation.^[^ ^23^, ^24^ ^]^ Recent studies identify fragile X mental retardation protein 1 (FMR1) as a novel m⁶A reader regulating RNA stability and function.^[^ ^25^, ^26^ ^]^ Although FMR1's function has been extensively characterized in the nervous system, its role as a m⁶A reader in cancer, particularly in ccRCC, remains largely unexplored.

Here, we delineate a novel ERβ/circAHNAK/FMR1/ADAM17 signaling axis. Mechanistically, ERβ upregulates circAHNAK expression, which competes with USP10 for binding to FMR1, thereby inhibiting USP10‐mediated deubiquitination and promoting FMR1 proteasomal degradation. FMR1 depletion impairs m⁶A‐modified mRNA recognition, consequently blocking METTL14‐dependent ADAM17 transcript degradation and stabilizing ADAM17 expression. Elevated ADAM17 levels are further enriched in extracellular vesicles derived from ccRCC cells, potentiating their pro‐angiogenic activity. Our findings uncover a previously unrecognized regulatory mechanism by which ERβ promotes angiogenesis and highlight a circRNA–deubiquitination–m⁶A interaction network as a new paradigm for tumor vascularization and therapeutic intervention.

## 2 Results

### 2.1 ERβ Upregulation Correlates with Poor Prognosis and Promotes Angiogenesis via circAHNAK in ccRCC

**Figure 1:**
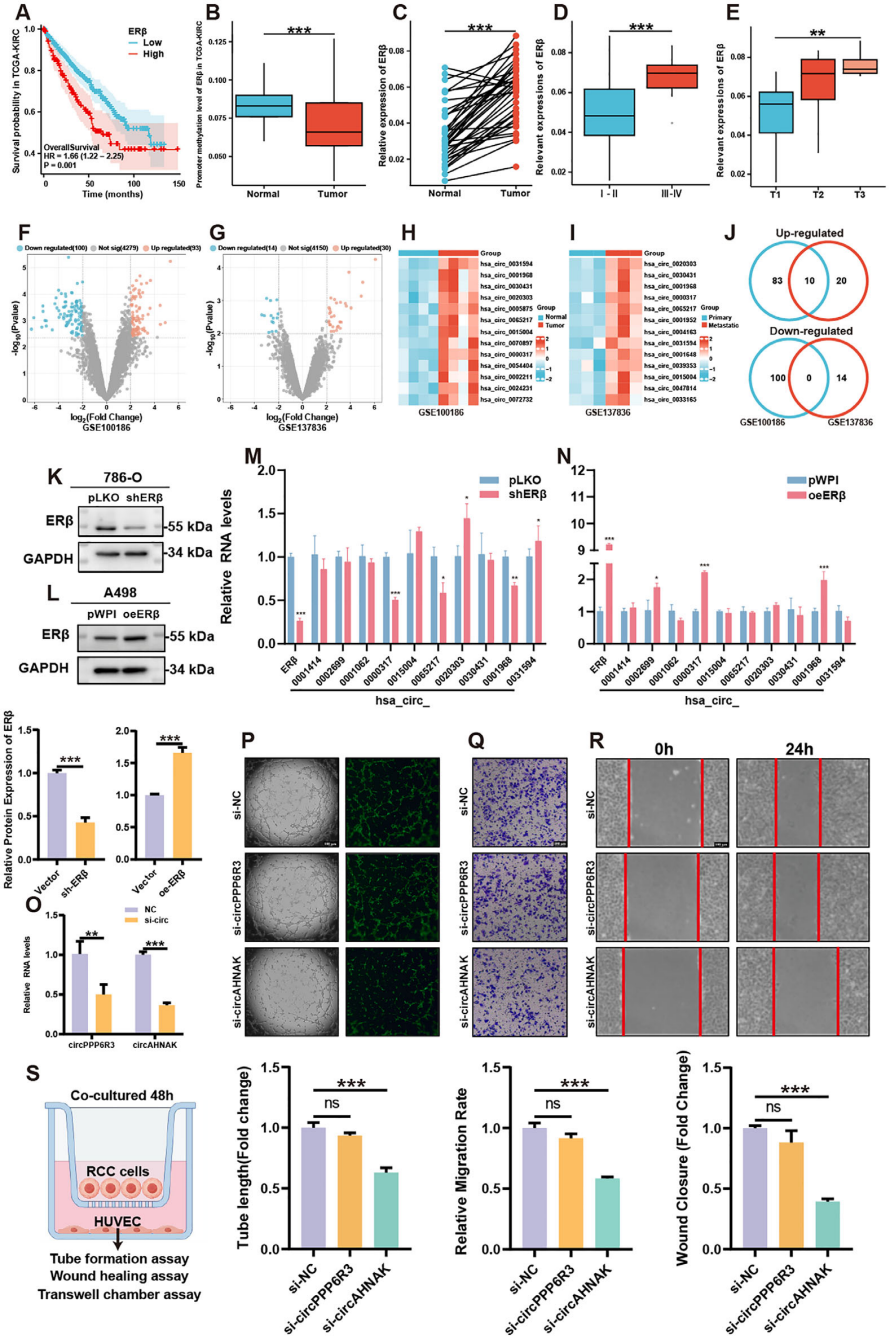
ERβ upregulation predicts poor prognosis and drives circAHNAK‐mediated angiogenesis in ccRCC. A) Kaplan–Meier OS analysis based on ERβ expression in the TCGA‐KIRC cohort. B) Promoter methylation levels of ERβ are reduced in TCGA‐KIRC tumors compared with normal tissues. C) ERβ expression is elevated in 38 paired ccRCC and adjacent normal tissues. D,E) High ERβ expression is positively correlated with advanced pathological grade and stage in ccRCC. (F, G) Volcano plots of differentially expressed circRNAs in ccRCC vs adjacent normal tissues (GSE100186) and metastatic vs primary ccRCC tumors (GSE137836) (|log_2_FC| > 2, p < 0.01). H,I) Heatmaps showing the top 15 upregulated circRNAs in each dataset; red and blue indicate upregulation and downregulation, respectively. J) Venn diagram identifying 10 commonly upregulated circRNAs across both datasets. (K, L) Western blot and quantification of ERβ knockdown in 786‐O cells and overexpression in A498 cells. M,N) Expression levels of selected circRNA candidates in response to ERβ modulation. circAHNAK (hsa_circ_0000317) and circPPP6R3 (hsa_circ_0001968) were positively regulated by ERβ. O) qRT‐PCR confirmation of siRNA‐mediated knockdown efficiency of circAHNAK and circPPP6R3. P–R) Functional assays in a ccRCC–HUVEC co‐culture system showing impaired tube formation, migration, and wound healing of HUVECs upon circAHNAK knockdown in 786‐O cells. Quantification is shown in bar graphs. S) Schematic of the tumor–endothelial co‐culture system. Data are presented as mean ± SEM of three independent experiments. ^*^ *P* < 0.05, ^**^ *P* < 0.01, ^***^ *P* < 0.001 vs control. Scale bar = 100 µm.

Clinical data from TCGA‐KIRC revealed that high ERβ expression was associated with shorter overall survival (OS), disease‐specific survival (DSS), and progression‐free interval (PFI) (**Figure** **1**A; Figure S1A,B, Supporting Information). Promoter methylation analysis further showed reduced methylation levels of ERβ in ccRCC tumors compared with normal tissues (Figure 1B). Consistently, ERβ expression was elevated in 38 paired tumor and adjacent normal samples (Figure 1C), and higher ERβ levels correlated with advanced pathological grade and stage (Figure 1D,E). These findings were further validated by immunohistochemistry in an independent cohort of 20 paired tissues (Figure S1C,D, Supporting Information). Collectively, these findings indicate that ERβ is upregulated in ccRCC and correlates with poor prognosis.

Previous studies demonstrated that ERβ promotes angiogenesis in ccRCC and mediates tumor progression through circRNAs.^[^ ^9^, ^10^ ^]^ However, the mechanistic link between ERβ‐regulated circRNAs and ccRCC angiogenesis remains unclear. To identify functionally relevant circRNAs, two GEO microarray datasets were analyzed, GSE100186 (circRNA profiles in paired ccRCC and adjacent normal tissues) and GSE137836 (primary vs metastatic tumors). Differential expression analysis (*p* < 0.01, log2FC > 2) identified significantly altered circRNAs (Figure 1F,G), with heatmap visualization highlighting the top 15 upregulated candidates in each dataset (Figure 1H,I). Venn analysis revealed 10 circRNAs consistently upregulated across both datasets (Figure 1J). To evaluate their association with ERβ, we performed knockdown experiments in 786‐O cells (high ERβ expression) and overexpression in A498 cells (low ERβ expression) (Figure 1K,L; Figure S1E,F, Supporting Information). qRT‐PCR validation revealed two circRNAs, hsa_circ_0000317 and hsa_circ_0001968, whose expression levels tracked with ERβ status (Figure 1M,N).

To examine tumor–endothelial interactions, a direct co‐culture system was established in which HUVECs were cultured with ccRCC cells for 48 h before functional assays (Figure 1S). Using this platform, angiogenesis assays, including tube formation, wound healing, and Transwell migration, were employed to assess the roles of candidate circRNAs. CircAHNAK (hsa_circ_0000317) knockdown in ccRCC cells markedly reduced their capacity to promote HUVEC tubulogenesis and motility, whereas silencing circPPP6R3 (hsa_circ_0001968) had no appreciable effect (Figure 1O–R). Cumulatively, these data establish circAHNAK as a critical mediator of ERβ‐driven angiogenesis in ccRCC.

### 2.2 Molecular and Functional Characterization of circAHNAK in ccRCC

**Figure 2:**
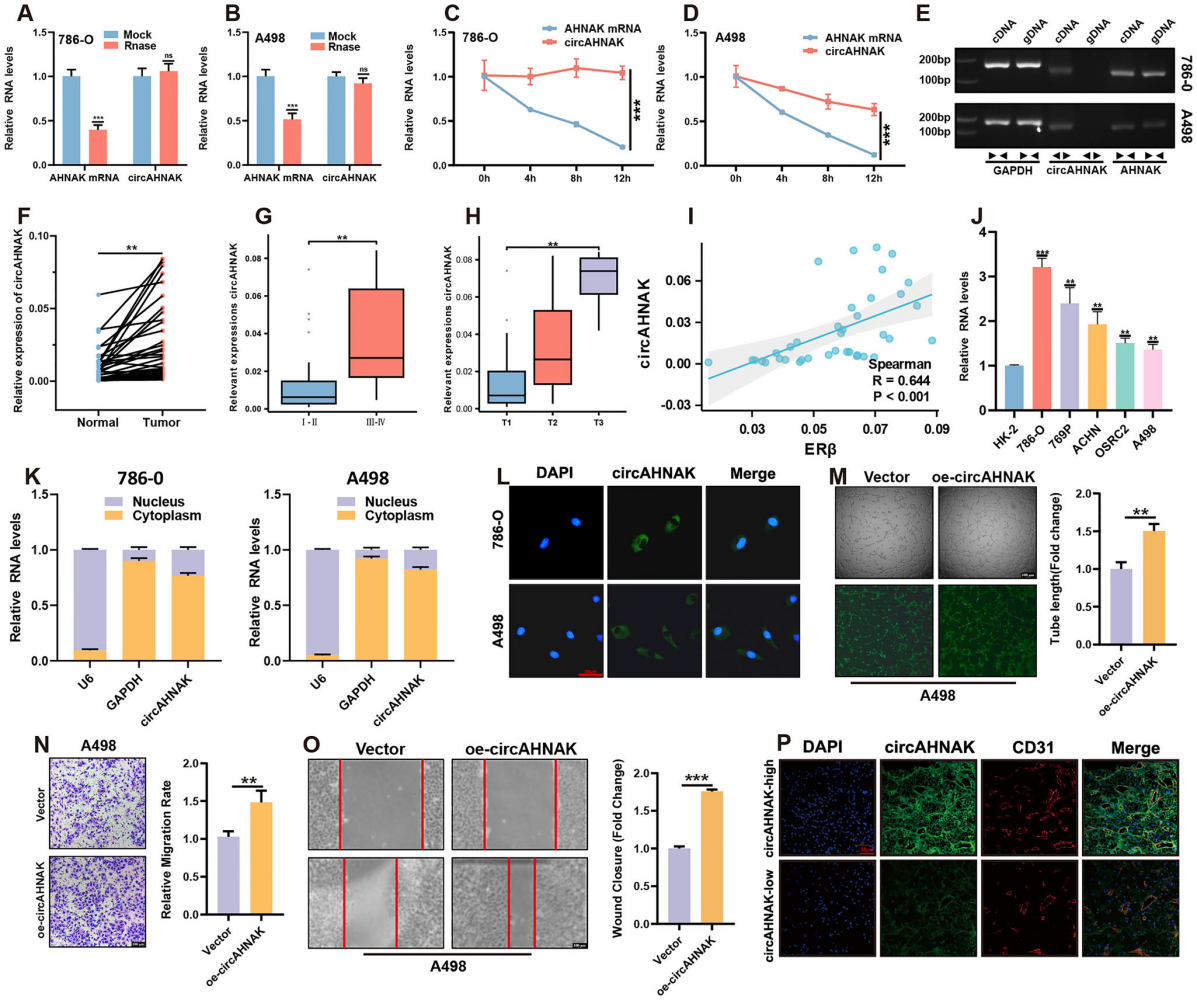
Molecular characterization and functional role of circAHNAK in ccRCC angiogenesis. A,B) RNase R(5 U/µg) treatment demonstrated that circAHNAK is resistant to exonuclease digestion compared to linear AHNAK mRNA. C,D) Actinomycin D(2 µg/mL) assay showed that circAHNAK exhibits greater stability than linear AHNAK transcripts over time. E) PCR analysis using divergent primers amplified circAHNAK only from cDNA, whereas convergent primers amplified linear AHNAK from both cDNA and genomic DNA, confirming the circular nature of circAHNAK. F) qRT‐PCR analysis of 38 paired ccRCC tumors and adjacent normal tissues revealed significant upregulation of circAHNAK in tumors. G,H) Elevated circAHNAK expression correlated with higher pathological grade and advanced stage. I) Correlation analysis indicated a positive association between ERβ and circAHNAK expression. J) Elevated circAHNAK expression was also observed in ccRCC cell lines compared to normal renal epithelial HK2 cells. K,L) Subcellular fractionation assays indicated predominant cytoplasmic localization of circAHNAK in ccRCC cells, which was further confirmed by RNA FISH. M–O) Functional assays using a ccRCC–HUVEC co‐culture system showed that circAHNAK overexpression in A498 cells enhanced HUVEC tube formation and migration. P) IF staining of ccRCC tumor tissues revealed a positive correlation between circAHNAK expression and microvessel density. Data are presented as mean ± SEM from at least three independent experiments. ^*^ *P* < 0.05, ^**^ *P* < 0.01, ^***^ *P* < 0.001 vs control. Scale bars = 100 µm (M–O) or 50 µm (L,P).

To confirm the circular structure of circAHNAK, RNase R digestion assays were performed, demonstrating resistance to exonuclease‐mediated degradation (**Figure** **2**A,B). Actinomycin D treatment further revealed that circAHNAK exhibits significantly greater stability than its linear AHNAK mRNA counterpart (Figure 2C,D). Using divergent primers specific for back‐splice junctions and convergent primers targeting linear transcripts, circAHNAK was amplified exclusively from cDNA, whereas linear AHNAK was detected in both cDNA and genomic DNA (Figure 2E), validating circAHNAK as a stable circular RNA species in ccRCC.

In 38 paired ccRCC samples, qRT‐PCR revealed significantly higher circAHNAK expression in tumor tissues compared with adjacent normal tissues (Figure 2F). Elevated circAHNAK expression was closely associated with higher pathological grade and advanced stage (Figure 2G,H). Correlation analysis further revealed a significant positive relationship between ERβ and circAHNAK levels (Figure 2I). Consistently, several ccRCC cell lines exhibited markedly elevated circAHNAK expression compared to normal renal tubular epithelial HK2 cells (Figure 2J). Subcellular fractionation combined with RNA fluorescence in situ hybridization (FISH) revealed predominant cytoplasmic localization of circAHNAK (Figure 2K,L).

To assess the functional role of circAHNAK in ccRCC angiogenesis, stable circAHNAK knockdown was established in 786‐O cells (sh‐circAHNAK) and overexpression in A498 cells (oe‐circAHNAK). In a direct co‐culture system with HUVECs, circAHNAK depletion significantly impaired endothelial tube formation and migration, whereas overexpression enhanced these angiogenic activities (Figure 2M–O; Figure S2A–C, Supporting Information). Colony formation, CCK‐8, and EdU assays confirmed that circAHNAK modulation in ccRCC cells also influences HUVEC proliferation, supporting its pro‐angiogenic role (Figure S2D–F, Supporting Information). Because endothelial migration is a key initiating and rate‐limiting step in tumor angiogenesis, subsequent experiments focused on migration and tube formation to directly investigate circAHNAK‐mediated angiogenic mechanisms. Immunofluorescence (IF) staining of tumor tissues further confirmed a positive correlation between circAHNAK expression levels and microvessel density (Figure 2P).

Together, these data establish circAHNAK as a stable and upregulated circular RNA in ccRCC that promotes tumor angiogenesis.

### 2.3 ERβ Transcriptionally Regulates circAHNAK Expression

**Figure 3:**
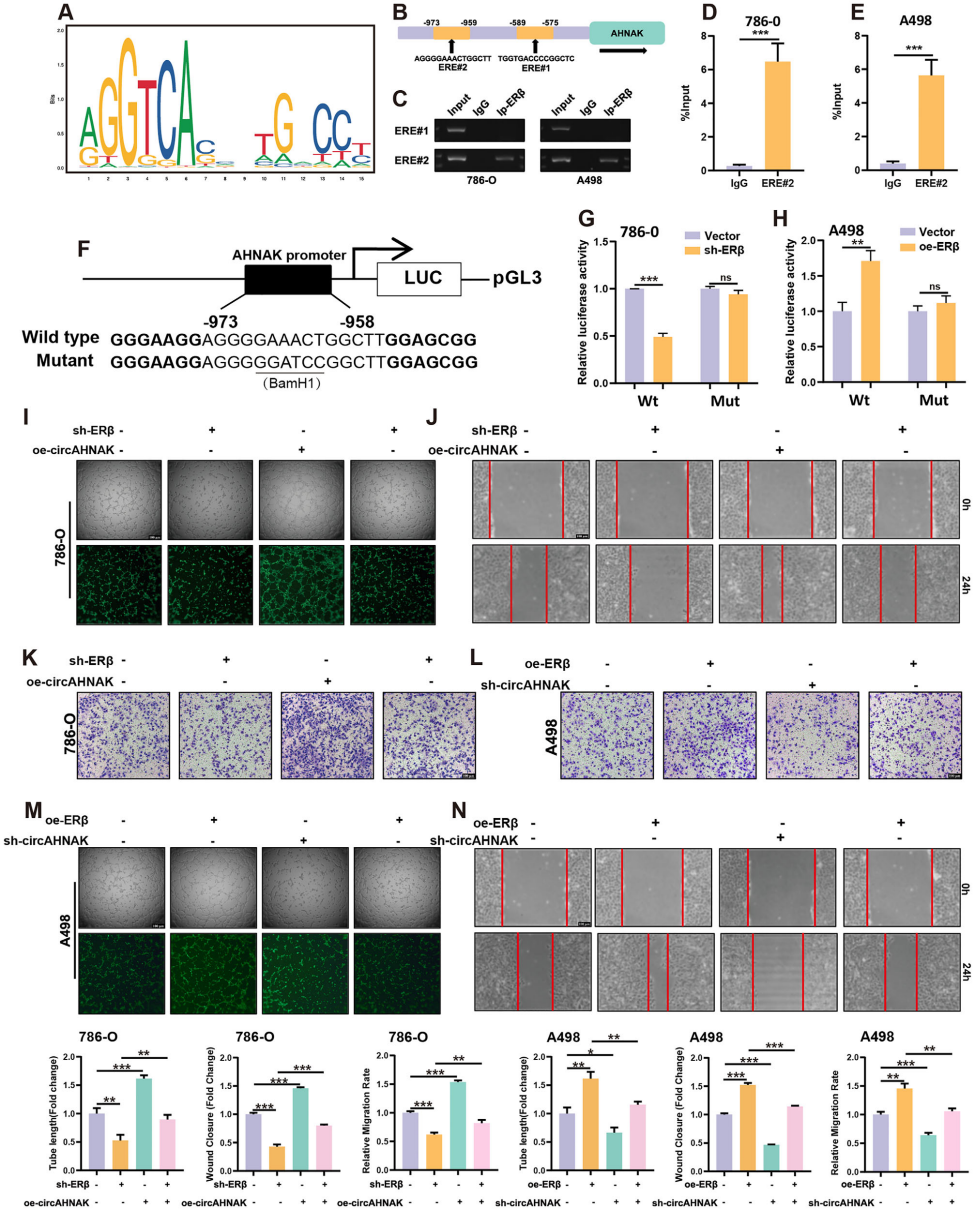
ERβ transcriptionally upregulates circAHNAK via direct binding to the AHNAK promoter. A) Schematic representation of the ERβ binding motif. B) Two putative EREs in the AHNAK promoter region were identified using the JASPAR database. C–E) ChIP‐qPCR analysis confirmed that ERβ specifically binds to ERE#2, but not ERE#1, in the AHNAK promoter in both 786‐O and A498 cells. F) Diagram of luciferase reporter constructs containing Wt or ERE#2‐Mut AHNAK promoter sequences. G,H) Dual‐luciferase reporter assays showed that ERβ knockdown in 786‐O cells significantly reduced Wt promoter activity, whereas ERβ overexpression in A498 cells enhanced it. These effects were abolished in the Mut construct, indicating transcriptional regulation through ERE#2. I–K) Functional rescue experiments in the 786‐O–HUVEC co‐culture system demonstrated that circAHNAK overexpression restored the impaired HUVEC tube formation and migration caused by ERβ knockdown. L–N) Conversely, circAHNAK silencing abrogated the enhanced angiogenic effects induced by ERβ overexpression in A498–HUVEC co‐cultures. Bar graphs show the quantification of each assay. Data are presented as mean ± SEM from three independent experiments. ^*^ *P* < 0.05, ^**^ *P* < 0.01, ^***^ *P* < 0.001 vs control. Scale bar = 100 µm.

To elucidate the molecular mechanism underlying ERβ‐mediated transcriptional regulation of circAHNAK, the promoter region of AHNAK, the host gene of circAHNAK, was analyzed using the JASPAR database.^[^ ^27^ ^]^ This analysis identified two putative estrogen response elements (EREs) within a 2‐kb region upstream of the transcription start site (**Figure** **3**A,B). Chromatin immunoprecipitation followed by qPCR (ChIP‐qPCR) confirmed that ERβ specifically binds to ERE#2 in both 786‐O and A498 ccRCC cells (Figure 3C–E). To functionally validate this interaction, luciferase reporter plasmids containing either the wild‐type (Wt) AHNAK promoter or a mutant (Mut) promoter with disrupted ERE#2 sequences were constructed (Figure 3F). Luciferase assays showed that ERβ knockdown (shERβ) in 786‐O cells significantly reduced promoter activity only in the Wt construct, whereas ERβ overexpression (oeERβ) in A498 cells enhanced activity exclusively in Wt‐transfected cells (Figure 3G,H), confirming the specificity of ERβ’s transcriptional regulation via ERE#2. Further rescue experiments provided strong support for this regulatory axis. Specifically, oe‐circAHNAK abolished the inhibitory effects of ERβ silencing on HUVEC migration and tube formation in 786‐O co‐cultures (Figure 3I–K). Conversely, sh‐circAHNAK counteracted the pro‐angiogenic effects triggered by ERβ overexpression in A498 co‐cultures (Figure 3L–N).

Collectively, these results reveal that ERβ directly binds to ERE#2 in the AHNAK promoter to transcriptionally upregulate circAHNAK, which plays a pivotal role in mediating ERβ‐induced angiogenesis in ccRCC.

### 2.4 circAHNAK Drives ccRCC Angiogenesis Through ADAM17 Upregulation

**Figure 4:**
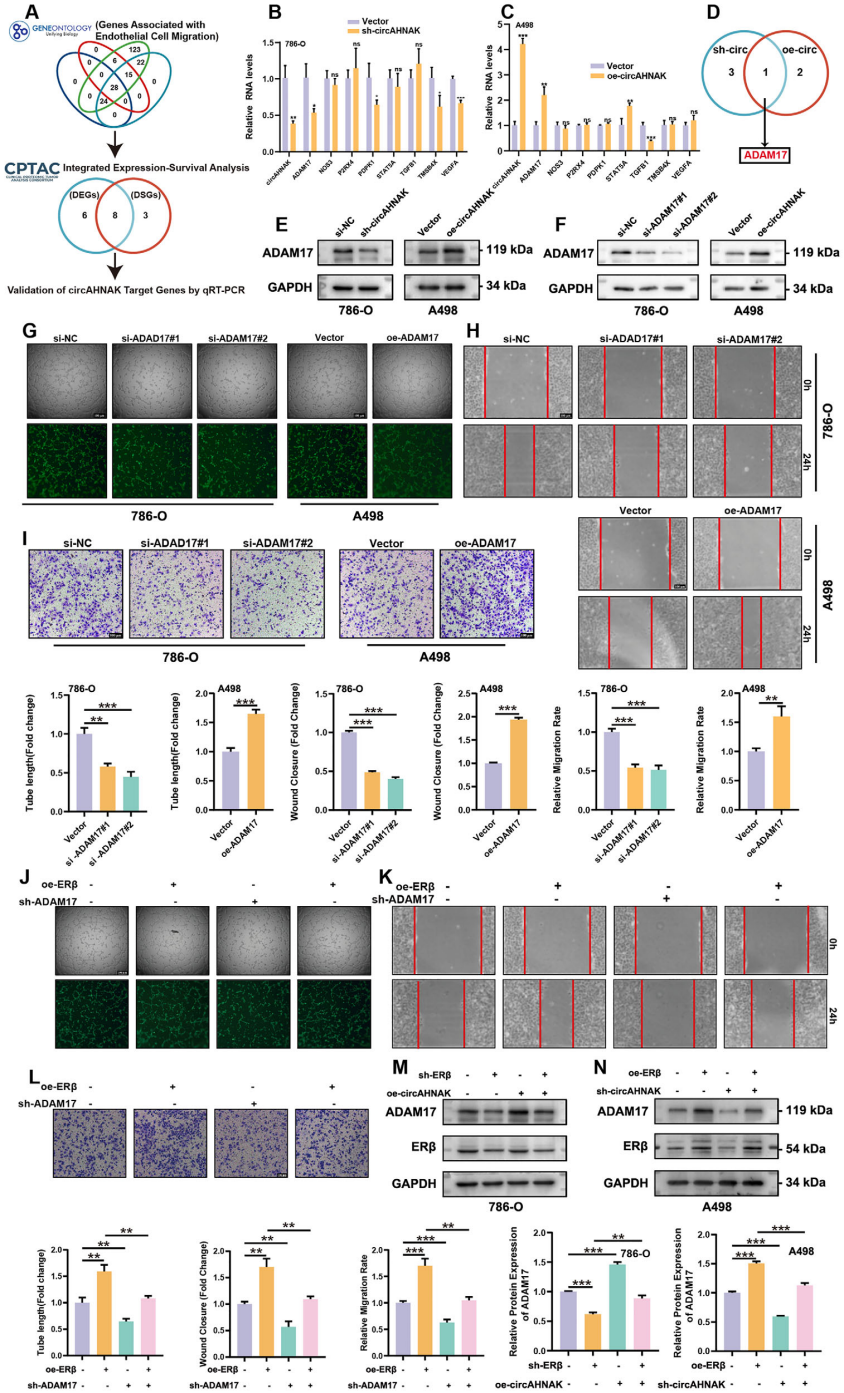
ADAM17 acts as a downstream effector of circAHNAK in promoting ccRCC angiogenesis. A) Schematic workflow illustrating the identification of angiogenesis‐related target genes using GO and CPTAC analyses. B–D) qRT‐PCR validation of candidate genes in 786‐O and A498 cells following circAHNAK knockdown or overexpression, respectively, identifying ADAM17 as a consistent downstream effector. E) Western blot analysis shows changes in ADAM17 protein levels in response to circAHNAK modulation. F) Validation of ADAM17 knockdown in 786‐O cells and overexpression in A498 cells by Western blot. G–I) Functional assays show that ADAM17 knockdown impairs, while its overexpression enhances, HUVEC tube formation and migration. Bar graphs show the quantification of each assay. J–L) Rescue experiments demonstrate that silencing ADAM17 reverses the pro‐angiogenic effects of ERβ overexpression in A498 cells. M,N) Western blot analysis shows that circAHNAK overexpression restores ADAM17 expression suppressed by ERβ knockdown in 786‐O cells, whereas circAHNAK silencing blocks ERβ‐induced ADAM17 upregulation in A498 cells. Data are presented as mean ± SEM from three independent experiments. ^*^ *P* < 0.05, ^**^ *P* < 0.01, ^***^ *P* < 0.001 vs control. Scale bar = 100 µm.

To elucidate the mechanism by which circAHNAK promotes angiogenesis in ccRCC, gene ontology (GO) and CPTAC database analyses were integrated to identify differentially expressed angiogenesis‐related genes associated with prognosis (**Figure** **4**A; Figure S3A–D, Supporting Information). Subsequent validation experiments revealed that circAHNAK knockdown in 786‐O cells significantly modulated the expression of ADAM17, PDPK1, TMSB4X, and VEGFA, whereas circAHNAK overexpression in A498 cells upregulated ADAM17 and TGFB1 (Figure 4B–D). Among these, ADAM17 emerged as the most robustly and consistently regulated target, and was therefore selected for further mechanistic investigation. In our cohort of 38 ccRCC patients, ADAM17 expression was significantly elevated in tumors and positively correlated with higher Fuhrman grade and advanced T stage (Figure S3E,F, Supporting Information). qRT‐PCR analysis of ccRCC tumor tissues revealed a strong positive correlation between circAHNAK and ADAM17 expression, as well as between ERβ and ADAM17 (Figure S3G,H, Supporting Information). These correlations were further confirmed in the TCGA‐KIRC cohort (Figure S3I, Supporting Information). Collectively, these findings identified ADAM17 as the principal downstream effector mediating circAHNAK‐driven angiogenesis in ccRCC.

Extensive literature has established ADAM17 as a pivotal pro‐angiogenic factor in tumor progression. ^[^ ^28^, ^29^, ^30^ ^]^ sh‐circAHNAK reduced both ADAM17 mRNA and protein levels in 786‐O cells, whereas oe‐circAHNAK increased ADAM17 expression in A498 cells (Figure 4E; Figure S4A,B, Supporting Information). Functional assays further demonstrated that ADAM17 depletion in 786‐O cells markedly impairs HUVEC migration and tube formation, while ADAM17 overexpression in A498 cells enhances these angiogenic processes (Figure 4F–I; Figure S4C–F, Supporting Information). Rescue experiments further reinforced this regulatory axis. In 786‐O cells with ERβ depletion, oe‐circAHNAK partially restored ADAM17 expression, while in A498 cells, sh‐circAHNAK attenuated the ERβ‐induced upregulation of ADAM17 (Figure 4M,N). Importantly, ADAM17 knockdown (shADAM17) markedly reduced the pro‐angiogenic effects driven by ERβ overexpression in A498 cells, as demonstrated by impaired tube formation, wound healing, and Transwell migration (Figure 4J–L).

Altogether, these results establish the ERβ/circAHNAK/ADAM17 signaling axis as a crucial mechanism driving angiogenesis in ccRCC.

### 2.5 CircAHNAK Targets the C‐Terminal Region of FMR1 to Control ADAM17 Stability

**Figure 5:**
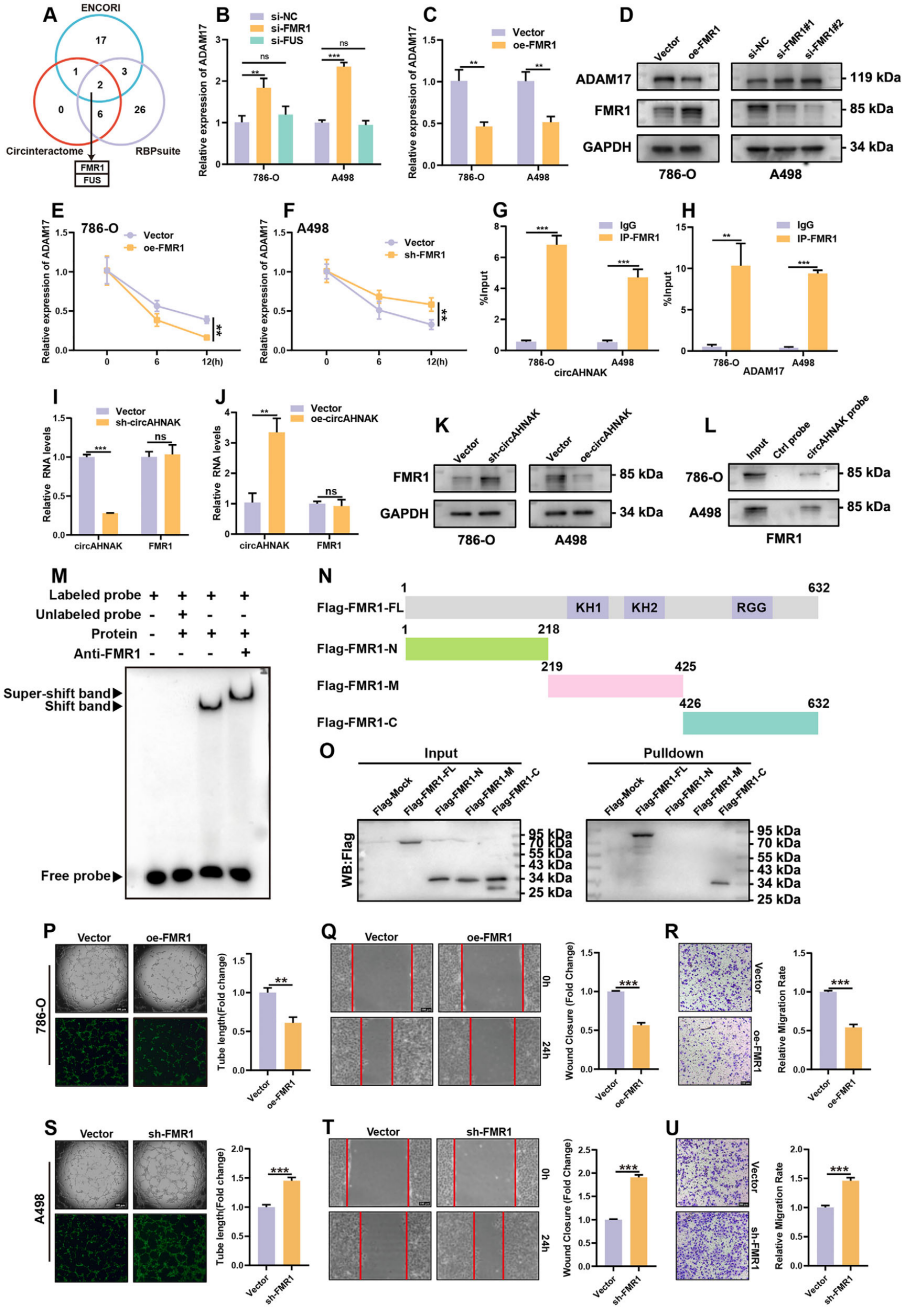
CircAHNAK interacts with FMR1 to regulate ADAM17 mRNA stability in ccRCC. A) Venn diagram showing the overlap of predicted circAHNAK‐binding RBPs from ENCORI, CircInteractome, and RBPsuite, identifying FMR1 and FUS as candidates. B) qRT‐PCR analysis shows that FMR1 knockdown increases ADAM17 mRNA levels in 786‐O and A498 cells, while FUS knockdown has no effect. C) qRT‐PCR analysis demonstrates that FMR1 overexpression significantly suppresses ADAM17 mRNA levels in both cell lines. D) Western blot analysis confirming FMR1‐mediated regulation of ADAM17 protein expression. E,F) Actinomycin D‐mediated (2 µg/mL) mRNA decay assays showed increased ADAM17 mRNA stability upon FMR1 knockdown and reduced stability upon FMR1 overexpression. G,H) RIP‐qPCR demonstrating enrichment of both circAHNAK and ADAM17 transcripts in FMR1 immunoprecipitates. I–K) Western blot and qRT‐PCR analyses show that circAHNAK regulates FMR1 protein expression without affecting its mRNA levels. L,M) RNA pull‐down and EMSA using biotin‐labeled circAHNAK probes confirm direct interaction with FMR1. N,O) FLAG‐tagged FMR1 truncation constructs (N‐terminal, middle, and C‐terminal; FLAG‐FMR1‐N/M/C) were generated, and RNA pull‐down assays verified that circAHNAK binds to the C‐terminal region of FMR1. P–U) Functional assays show that FMR1 knockdown enhances, while its overexpression suppresses, HUVEC tube formation and migration. Bar graphs show the quantification of each assay. All data are presented as mean ± SEM from three independent experiments. ^*^ *P* < 0.05, ^**^ *P* < 0.01, ^***^ *P* < 0.001 vs control. Scale bar = 100 µm.

To investigate the molecular mechanism by which circAHNAK promotes ccRCC angiogenesis through ADAM17, bioinformatic analyses were first performed to identify potential circAHNAK‐interacting proteins. Overlapping predictions from ENCORI, CircInteractome,^[^ ^31^ ^]^ and RBPsuite^[^ ^32^ ^]^ revealed only two candidate circAHNAK‐binding proteins, FMR1 and FUS (**Figure** **5**A). Knockdown of FMR1 or FUS, followed by assessment of ADAM17 expression, showed that only FMR1 depletion significantly increased ADAM17 mRNA levels in both 786‐O and A498 cells (Figure 5B; Figure S5A,B, Supporting Information). Conversely, FMR1 overexpression markedly reduced ADAM17 mRNA expression, consistent with Western blot validation (Figure 5C,D; Figure S5C,H,I, Supporting Information).

Previous studies have shown that FMR1 plays a critical role in determining mRNA fate in a tissue‐ and disease‐dependent manner and can directly regulate mRNA stability.^[^ ^25^, ^33^ ^]^ Notably, analysis of the TCGA‐KIRC cohort revealed that FMR1 is downregulated in tumors, and lower FMR1 expression is associated with poorer OS, PFI, and DSS (Figure S5D–G, Supporting Information). In line with this, actinomycin D chase assays demonstrated that FMR1 silencing prolonged ADAM17 mRNA half‐life, whereas FMR1 overexpression accelerated its decay (Figure 5E,F). RNA immunoprecipitation (RIP) assays further confirmed enrichment of FMR1 with both circAHNAK and ADAM17 transcripts in 786‐O and A498 cells (Figure 5G,H).

Examination of FMR1 expression following circAHNAK modulation revealed that circAHNAK knockdown or overexpression significantly altered FMR1 protein abundance without affecting transcript levels (Figure 5I–K; Figure S5J,K, Supporting Information). Importantly, RNA pull‐down and RNA‐EMSA assays using a biotinylated circAHNAK probe consistently confirmed that FMR1 is a direct binding protein of circAHNAK (Figure 5L,M). To determine the binding region, FLAG‐tagged FMR1 truncation constructs (N‐terminal, middle, and C‐terminal; FLAG‐FMR1‐N/M/C) were generated and subjected to both RNA pull‐down and RIP assays. RNA pull‐down demonstrated that circAHNAK specifically interacts with the C‐terminal region of FMR1, while RIP using the truncation constructs further confirmed that this region can enrich both circAHNAK and ADAM17 transcripts (Figure 5N,O; Figure S5L, Supporting Information). Functional angiogenesis assays demonstrated that FMR1 knockdown enhanced HUVEC migration and tubulogenesis, while FMR1 overexpression suppressed these effects (Figure 5P–U).

These data indicate that circAHNAK binds FMR1 via its C‐terminal region, suppressing FMR1 protein and regulating ADAM17 mRNA stability, thereby promoting ccRCC angiogenesis.

### 2.6 circAHNAK Regulates FMR1 Stability via the Ubiquitin–Proteasome Pathway in ccRCC

**Figure 6:**
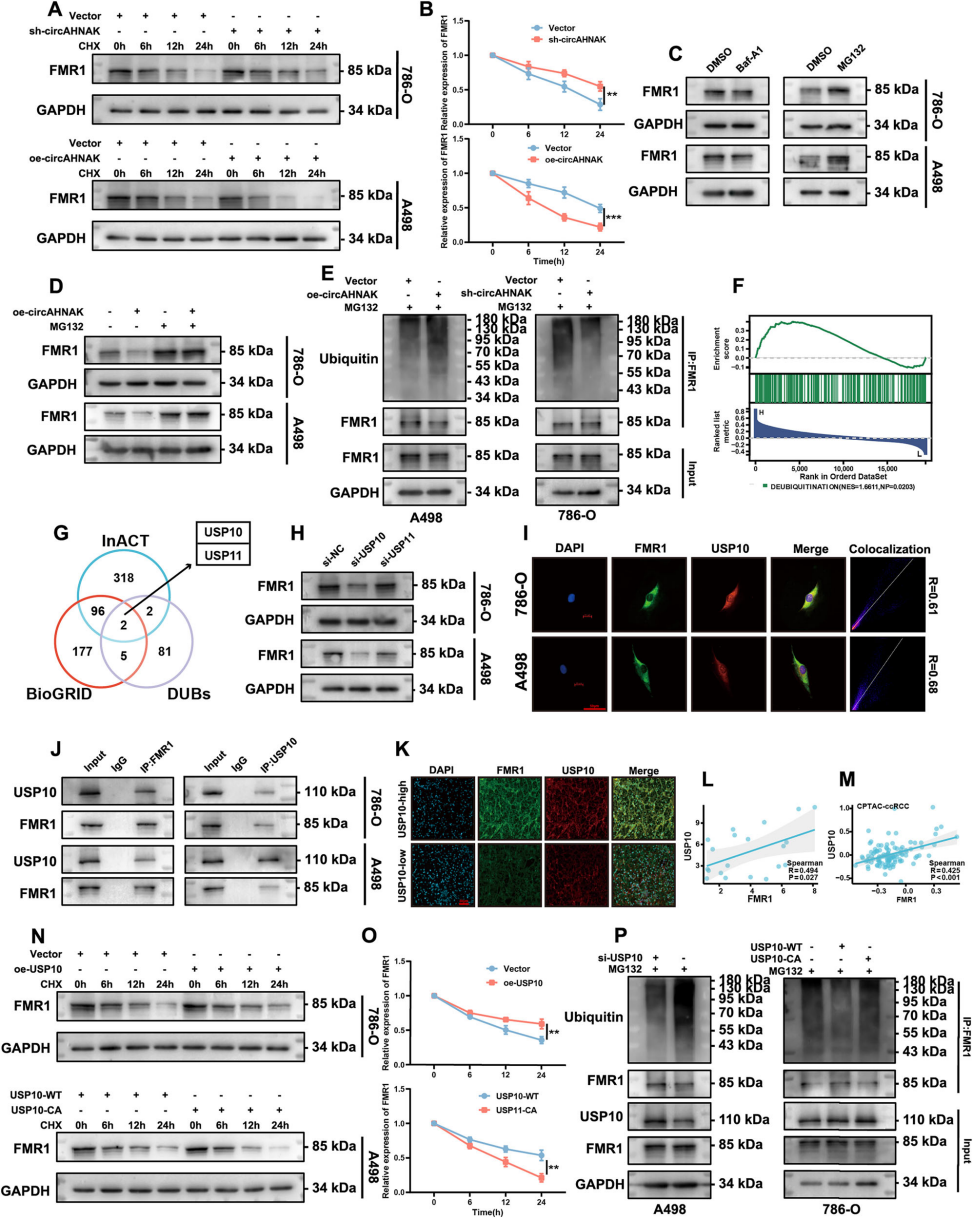
circAHNAK regulates FMR1 stability by modulating USP10‐mediated ubiquitination in ccRCC. A,B) Cycloheximide (CHX, 100 µg/mL) chase assays show that circAHNAK overexpression accelerates FMR1 protein degradation, whereas circAHNAK knockdown stabilizes FMR1 in ccRCC cells. C,D) Western blot analysis indicates that the proteasome inhibitor MG132 (10 µm, 8 h), but not the lysosomal inhibitor BafA1 (50 nm, 8 h), prevents FMR1 degradation and rescues FMR1 levels suppressed by circAHNAK overexpression. E) Ubiquitination assays demonstrate that circAHNAK knockdown reduces FMR1 ubiquitination, whereas circAHNAK overexpression enhances FMR1 ubiquitination. F) GSEA analysis of the TCGA‐KIRC dataset reveals significant enrichment of FMR1 in deubiquitination‐associated pathways. G) USP10 and USP11 were identified as candidate deubiquitinases (DUBs) for FMR1 through InACT and BioGRID databases. H) Western blot shows that USP10 knockdown, but not USP11 knockdown, decreases FMR1 protein levels, indicating that USP10 is the primary DUB for FMR1. I) IF staining demonstrates cytoplasmic colocalization of USP10 and FMR1 in 786‐O and A498 cells. J) Co‐IP confirms an endogenous interaction between USP10 and FMR1.K,L) IF staining of ccRCC tissues shows a positive correlation between USP10 and FMR1 expression. M) CPTAC proteomic data further confirm a positive correlation between USP10 and FMR1 protein levels in ccRCC. N,O) CHX assays show that USP10 overexpression protects FMR1 from degradation, whereas the catalytically inactive mutant USP10‐CA reverses this effect. P) Ubiquitination assays show that USP10 knockdown increases FMR1 ubiquitination, whereas wild‐type USP10 (USP10‐WT) decreases it, while the catalytically inactive mutant (USP10‐CA) fails to reduce ubiquitination. Data are presented as mean ± SEM from three independent experiments. ^*^ *P* < 0.05, ^**^ *P* < 0.01, ^***^ *P* < 0.001 vs control. Scale bar = 50 µm.

Given that circAHNAK alters FMR1 protein abundance without affecting its transcript levels, its impact on FMR1 protein stability was examined. Cycloheximide (CHX) chase assays demonstrated that circAHNAK overexpression accelerated FMR1 degradation, whereas circAHNAK knockdown markedly stabilized FMR1 (**Figure** **6**A,B). Treatment with the proteasome inhibitor MG132, but not the lysosomal inhibitor BafA1, prevented FMR1 degradation and restored its expression in circAHNAK‐overexpressing cells (Figure 6C,D; Figure S6A–D, Supporting Information). Ubiquitination assays further confirmed that circAHNAK knockdown reduced, whereas circAHNAK overexpression enhanced, FMR1 ubiquitination (Figure 6E), indicating that circAHNAK promotes FMR1 degradation via the ubiquitin‐proteasome pathway.

Gene Set Enrichment Analysis (GSEA) of TCGA‐KIRC data revealed significant enrichment of FMR1 in deubiquitination‐related pathways (Figure 6F). Bioinformatic analyses using IntAct^[^ ^34^ ^]^ and BioGRID^[^ ^35^ ^]^ databases identified USP10 and USP11 as candidate deubiquitinases interacting with FMR1 (Figure 6G). Functional screening confirmed that USP10, but not USP11, positively regulates FMR1 protein stability in both 786‐O and A498 cells (Figure 6H). IF staining demonstrated cytoplasmic co‐localization of USP10 and FMR1 in ccRCC cells (Figure 6I), which was further supported by Co‐IP (Co‐immunoprecipitation) assays confirming an endogenous USP10–FMR1 interaction (Figure 6J). Consistently, immunostaining of ccRCC tissues and CPTAC proteomic analysis showed a positive correlation between USP10 and FMR1 protein expression (Figure 6K–M).

To determine the dependence on USP10's catalytic activity, a catalytically inactive mutant (USP10‐CA) was employed. CHX assays revealed that USP10 overexpression protected FMR1 from degradation, whereas the catalytically inactive mutant USP10‐CA failed to exert this effect (Figure 6N,O). Ubiquitination assays further demonstrated that USP10 knockdown increased FMR1 ubiquitination, while wild‐type USP10 (USP10‐WT), but not USP10‐CA, effectively decreased it (Figure 6P). Together, these data demonstrate that circAHNAK enhances FMR1 ubiquitination and degradation via the ubiquitin‐proteasome pathway, and identify USP10 as the primary deubiquitinase of FMR1.

### 2.7 circAHNAK Competitively Interferes with USP10‐Mediated FMR1 Deubiquitination

**Figure 7:**
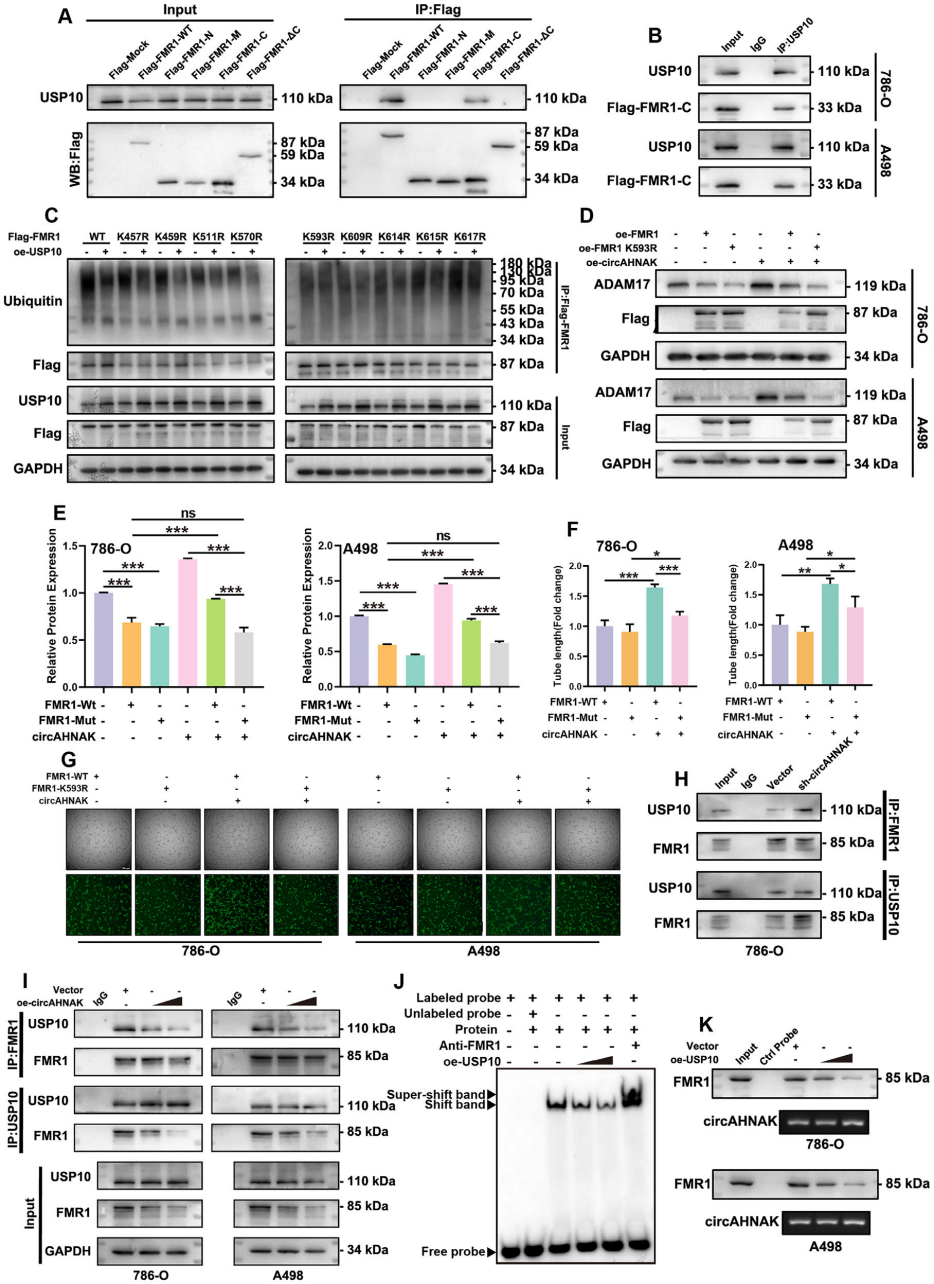
USP10 deubiquitinates FMR1 at K593, whereas circAHNAK interferes by competing for FMR1 binding. A) A series of truncated including Flag‐FMR1‐N, ‐M, ‐C, and ‐ΔC, was generated. Co‐IP assays with anti‐Flag antibody revealed that only Flag‐FMR1‐FL and Flag‐FMR1‐C interacted with USP10, whereas deletion of the C‐terminal region (ΔC) abolished this interaction. B) Reciprocal Co‐IP confirmed that USP10 directly binds the C‐terminal region of FMR1. C) Site‐directed mutagenesis identified lysine 593 (K593) as the major site of USP10‐mediated deubiquitination on FMR1. D,E) The K593R mutant exhibited resistance to circAHNAK‐induced degradation. F,G) Functional assays using a HUVEC co‐culture system demonstrated that the K593R mutant attenuated circAHNAK‐driven angiogenesis. H,I) Co‐IP assays further showed that circAHNAK knockdown enhanced the FMR1–USP10 interaction, whereas circAHNAK overexpression gradually weakened this binding. J,K) RNA‐EMSA and RNA pull‐down assays with biotin‐labeled circAHNAK probes confirmed the competitive relationship, showing that increasing USP10 expression significantly suppressed the interaction between circAHNAK and FMR1. Data are presented as mean ± SEM from three independent experiments. ^*^ *P* < 0.05, ^**^ *P* < 0.01, ^***^ *P* < 0.001 vs control. Scale bars = 100 µm.

To further define the molecular mechanism, Co‐IP experiments were performed using a series of truncated Flag‐FMR1 constructs. The results revealed that USP10 binds specifically to the C‐terminal region, as both full‐length FMR1 and Flag‐FMR1‐C interacted with USP10, whereas deletion of the C‐terminal region (ΔC) abolished this interaction (**Figure** **7**A,B). Site‐directed mutagenesis of lysine residues within this region identified lysine 593 (K593) as the primary site of USP10‐mediated deubiquitination (Figure 7C). Notably, the K593R mutant exhibited partial resistance to circAHNAK‐induced degradation, which led to reduced ADAM17 expression (Figure 7D,E). Functional angiogenesis assays, including tube formation, wound healing, and Transwell migration, showed that FMR1‐K593R partially reversed the pro‐angiogenic effects of circAHNAK overexpression in HUVECs (Figure 7F,G; Figure S6G–I, Supporting Information).

Given that both circAHNAK and USP10 bind to the C‐terminal region of FMR1, we hypothesized that circAHNAK might competitively interfere with USP10 binding. Consistent with reports that circRNAs can disrupt protein–protein interactions competitively,^[^ ^36^, ^37^ ^]^ Co‐IP assays showed that circAHNAK knockdown enhanced, while increasing circAHNAK expression progressively weakened the USP10–FMR1 interaction (Figure 7H,I; Figure S6J–L, Supporting Information). Similarly, RNA‐EMSA and RNA pull‐down assays confirmed that increasing USP10 expression dose‐dependently suppressed the circAHNAK–FMR1 interaction (Figure 7J,K; Figure S6M–O, Supporting Information). Molecular docking further suggested that USP10 interacts with Asp‐583, Asn‐586, and Thr‐592, whereas circAHNAK binds Thr‐592 and Ser‐589. The overlap at Thr‐592 indicates a potential competitive binding site, further supporting our experimental results (Figure S6P,Q, Supporting Information). Collectively, these findings establish a novel mechanism whereby USP10 deubiquitinates FMR1 at K593 to maintain its stability, whereas circAHNAK destabilizes FMR1 by competitively disrupting USP10 binding, thereby modulating ADAM17 expression and enhancing angiogenesis in ccRCC.

### 2.8 FMR1 Recognizes METTL14‐Dependent m⁶A Modification to Mediate ADAM17 mRNA Decay in ccRCC

**Figure 8:**
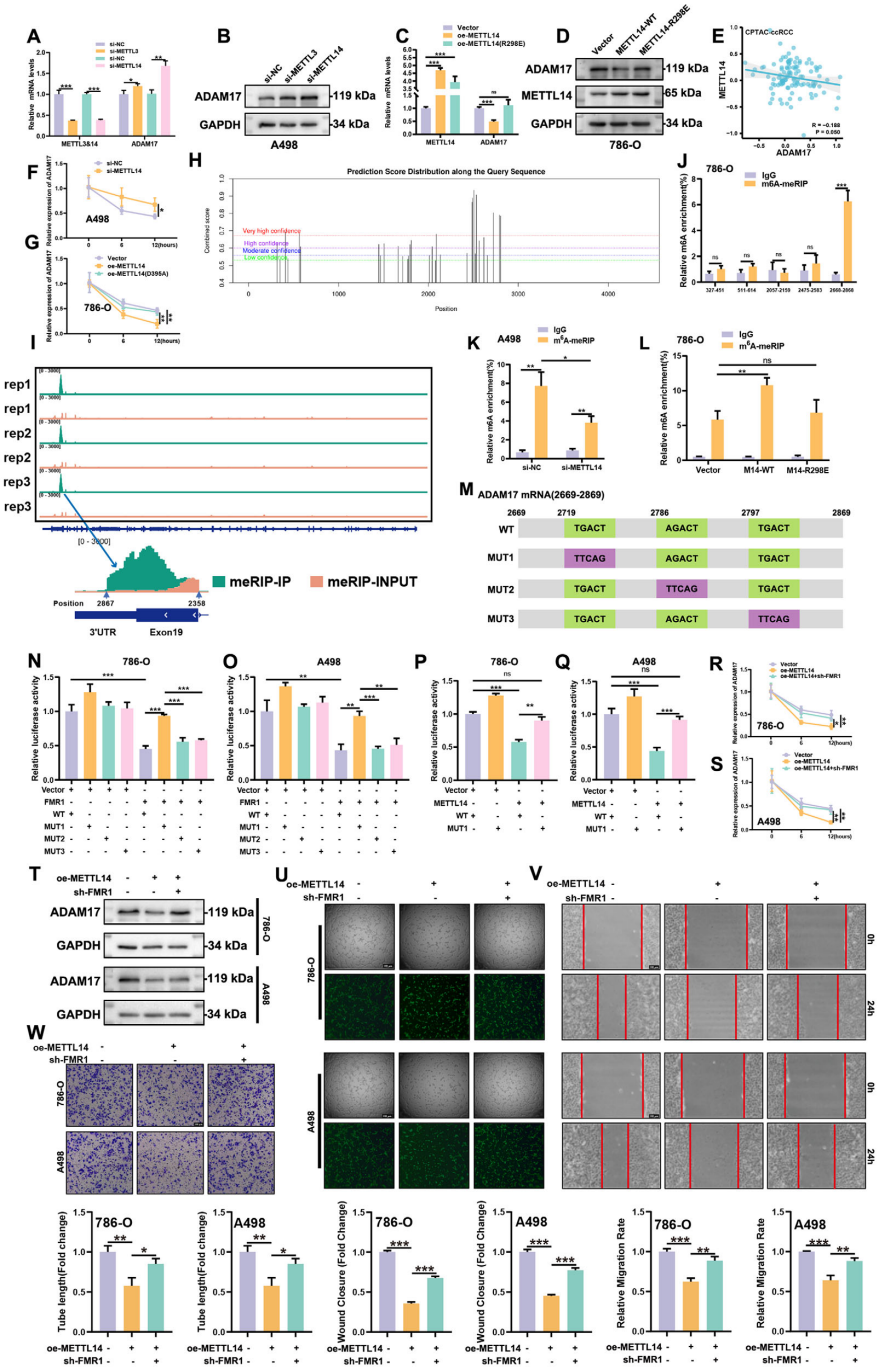
FMR1 recognizes METTL14‐dependent m⁶A modification to mediate ADAM17 mRNA decay in ccRCC. A,B) qRT‐PCR and Western blot analyses show that METTL14 knockdown increases ADAM17 expression more significantly than METTL3 knockdown in A498 cells. C,D) Overexpression of wild‐type METTL14, but not the catalytically inactive mutant (R298E), reduces ADAM17 levels in 786‐O cells. E) Analysis of the CPTAC dataset reveals a negative correlation between METTL14 and ADAM17 protein expression in ccRCC samples. F,G) Actinomycin D chase assays demonstrate that METTL14 depletion prolongs, whereas METTL14 overexpression shortens, ADAM17 mRNA half‐life in a catalytic activity–dependent manner. H,I) m⁶A‐meRIP‐seq and SRAMP prediction identify high‐confidence m⁶A peaks within the coding sequence and 3′UTR of ADAM17 mRNA. J–L) MeRIP‐qPCR confirms that the 2668–2868 region represents the primary METTL14‐dependent m⁶A‐modified site on ADAM17 mRNA. M–Q) Luciferase reporter assays with RRACH→TTCAG mutations at predicted m⁶A sites identify A2721 (MUT1) as essential for FMR1‐ and METTL14‐mediated repression. R–T) mRNA stability assays and Western blotting show that FMR1 knockdown rescues ADAM17 from METTL14‐induced degradation. U–W) Functional angiogenesis assays demonstrate that FMR1 silencing abrogates the anti‐angiogenic effects of METTL14 overexpression in HUVECs. Data are mean ± SEM from at least three independent experiments. ^*^ *P* < 0.05, ^**^ *P* < 0.01, ^***^ *P* < 0.001 vs control. Scale bar = 100 µm.

FMR1 has been shown to act as a m⁶A reader, facilitating the degradation of m⁶A‐modified transcripts by recruiting RNA decay machinery.^[^ ^25^, ^26^ ^]^ To examine whether FMR1 regulates ADAM17 mRNA stability in a m⁶A‐dependent manner, METTL3 or METTL14, core components of the m⁶A methyltransferase complex, were individually knocked down in A498 cells. METTL14 depletion led to a greater increase in ADAM17 levels than METTL3 knockdown (**Figure** **8**A,B; Figure S7A, Supporting Information). Although METTL3 knockdown caused a larger reduction in global m⁶A levels, MeRIP‐qPCR revealed that METTL14 depletion produced a more pronounced decrease in m⁶A enrichment specifically at the ADAM17 3′UTR, indicating a transcript‐ and site‐selective role for METTL14, prompting us to focus on METTL14 for subsequent mechanistic studies of ADAM17 regulation (Figure S7E–G, Supporting Information). Overexpression of wild‐type METTL14, but not its catalytically inactive R298E mutant, reduced ADAM17 expression in 786‐O cells (Figure 8C,D; Figure S7B, Supporting Information), consistent with the inverse correlation observed in CPTAC datasets (Figure 8E). Actinomycin D chase assays further confirmed that METTL14 knockdown stabilized ADAM17 mRNA, whereas METTL14 overexpression reduced its stability in a catalytic activity‐dependent manner, as the R298E mutant failed to exert this destabilizing effect (Figure 8F,G). m⁶A‐meRIP‐seq and in silico prediction using the SRAMP database (http://www.cuilab.cn/sramp/) revealed high‐confidence m⁶A peaks within the ADAM17 coding sequence and 3′UTR (Figure 8H,I).^[^ ^38^ ^]^ Primers targeting five representative regions were designed, and MeRIP‐qPCR identified the 2668–2868 region as the major METTL14‐dependent m⁶A‐modified site (Figure 8J–L). These data support the role of METTL14 as a key mediator of ADAM17 mRNA methylation.

Within the m⁶A‐enriched region, three putative m⁶A sites (A2721, A2788, A2799) with the highest confidence scores were further investigated. Luciferase reporter assays containing RRACH→TTCAG mutations at each site revealed that only the A2721 mutation (MUT1) abolished both FMR1‐ and METTL14‐mediated repression (Figure 8M–Q), pinpointing this site as functionally essential. Importantly, FMR1 knockdown rescued ADAM17 mRNA from METTL14‐induced degradation (Figure 8R–T; Figure S7C,D, Supporting Information). Finally, tube formation, wound healing, and Transwell migration demonstrated that the anti‐angiogenic effects of METTL14 overexpression in HUVECs were abolished upon FMR1 silencing (Figure 8U–W). Together, these results establish a novel mechanism whereby METTL14‐mediated m⁶A modification of ADAM17 mRNA is recognized by FMR1 to promote transcript decay. This post‐transcriptional regulation is critical for suppressing ADAM17‐driven angiogenesis in ccRCC.

### 2.9 Exosomal ADAM17 as a Key Mediator of ERβ‐Driven Angiogenesis in ccRCC

**Figure 9:**
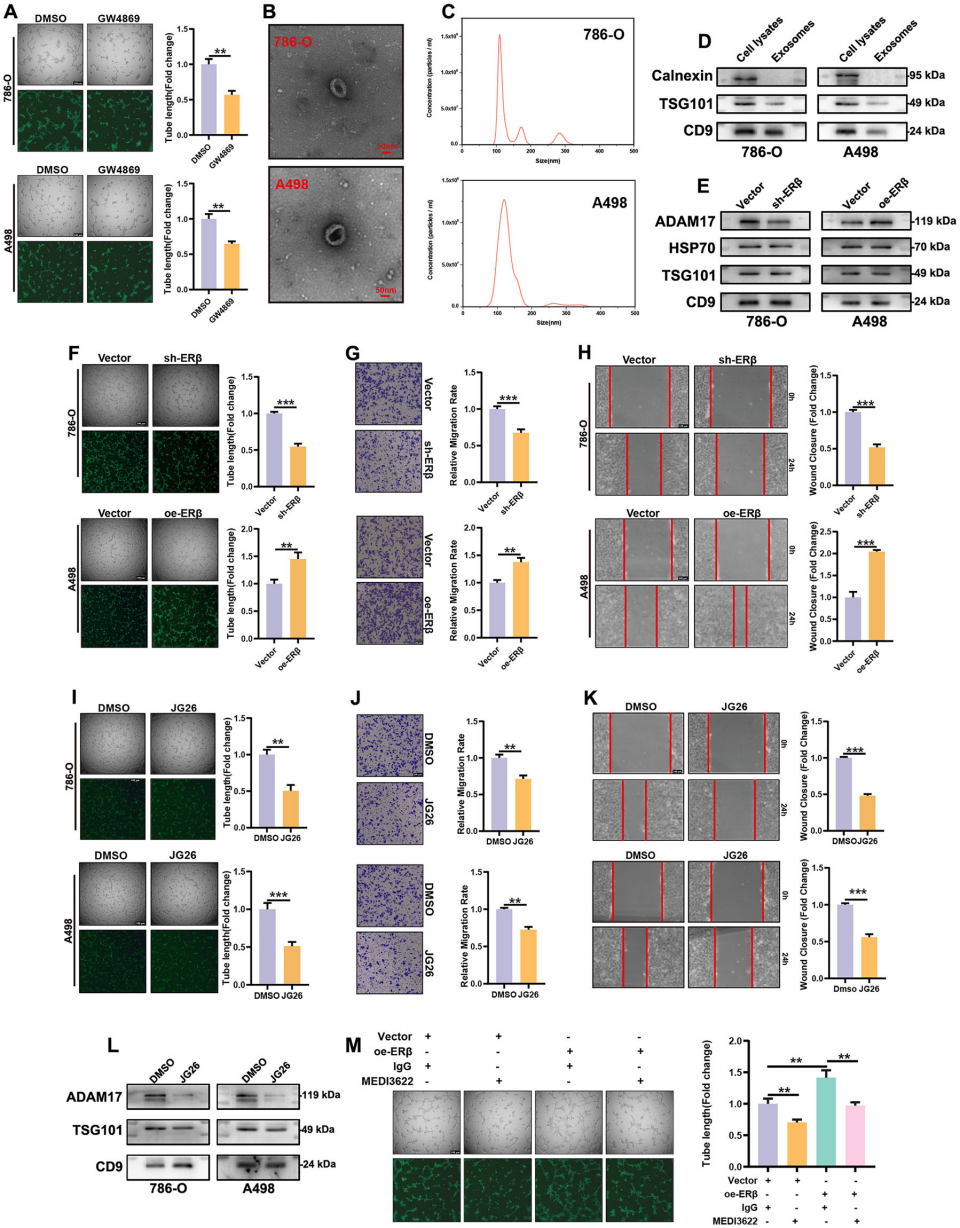
Exosomal ADAM17 mediates ERβ‐driven angiogenesis in ccRCC. A) GW4869 (10 µm, 24 h) inhibition of exosome release from 786‐O and A498 cells significantly reduced HUVEC tube formation. B,C) Characterization of exosomes from 786‐O and A498 cells by TEM (cup‐shaped morphology, scale bar = 50 nm) and NTA (≈100 nm diameter). D) Western blot confirmed exosomal markers TSG101 and CD9 and the absence of calnexin. E) ERβ knockdown decreased, whereas ERβ overexpression increased, ADAM17 levels in exosomes. F–H) Exosomes (10 µg/mL, 24 h) from ERβ‐overexpressing cells enhanced, while those from ERβ‐depleted cells impaired, HUVEC angiogenesis (tube formation, wound healing, migration; scale bar = 100 µm). I–K) Exosomes from JG26‐treated (25 µm, 24 h) RCC cells exhibited reduced pro‐angiogenic activity in HUVECs. L) Pretreatment with the ADAM17 inhibitor JG26 decreased ADAM17 levels in RCC cell‐derived exosomes. M) Neutralization of exosomal ADAM17 using the MEDI3622 antibody (10 µg /mL, 1 h) significantly reduced angiogenesis and reversed the pro‐angiogenic effect of ERβ‐overexpressing exosomes compared with the IgG control. Data are mean ± SEM from at least three independent experiments. ^*^ *P* < 0.05, ^**^ *P* < 0.01, ^***^ *P* < 0.001 vs control. Scale bar = 100 µm.

ADAM17 is traditionally recognized for its pro‐angiogenic activity via metalloproteinase‐dependent shedding of membrane‐bound substrates.^[^ ^29^, ^30^ ^]^ Recent studies suggest that tumor‐derived exosomes can serve as alternative carriers of functional ADAM17 to modulate endothelial cell behavior.^[^ ^39^ ^]^ Inhibition of exosome release from 786‐O and A498 cells using GW4869 (10 µm, 24 h) significantly reduced HUVEC tube formation, indicating a substantial role of exosomes in angiogenesis (**Figure** **9**A).

Exosomes isolated from 786‐O and A498 conditioned media exhibited the expected cup‐shaped morphology under transmission electron microscopy (scale bar = 50 nm) and an average diameter of ≈100 nm according to nanoparticle tracking analysis (Figure 9B,C). Western blotting confirmed the presence of canonical exosomal markers TSG101 and CD9 and the absence of the endoplasmic reticulum protein calnexin (Figure 9D). ERβ knockdown in 786‐O cells significantly decreased, whereas ERβ overexpression in A498 cells increased ADAM17 content in exosomes (Figure 9E; Figure S8A, Supporting Information). Functionally, HUVECs treated with exosomes from ERβ‐overexpressing cells displayed enhanced tube formation, wound closure, and migration, while exosomes from ERβ‐depleted cells impaired these angiogenic behaviors (Figure 9F–H).

Pretreatment of 786‐O and A498 cells with the ADAM17 inhibitor JG26 (25 µm, 24 h) before exosome isolation markedly reduced exosomal ADAM17 levels (Figure 9L; Figure S8B, C, Supporting Information), and these ADAM17‐deficient exosomes exhibited diminished pro‐angiogenic activity in HUVECs (Figure 9I–K). Additionally, neutralization of exosomal ADAM17 using the MEDI3622 antibody (10 µg/mL, 1 h) significantly decreased angiogenesis and reversed the pro‐angiogenic effect of exosomes from ERβ‐overexpressing cells compared with the IgG control (Figure 9M).

Collectively, these results demonstrate that exosomal ADAM17 is a critical effector of ERβ‐driven angiogenesis in ccRCC. This non‐canonical mechanism underscores the importance of tumor‐derived extracellular vesicles in modulating the tumor microenvironment and suggests a potential therapeutic avenue for targeting angiogenesis in ccRCC.

### 2.10 Targeting the ERβ/circAHNAK/FMR1/ADAM17 Axis Suppresses ccRCC Progression In Vivo

**Figure 10:**
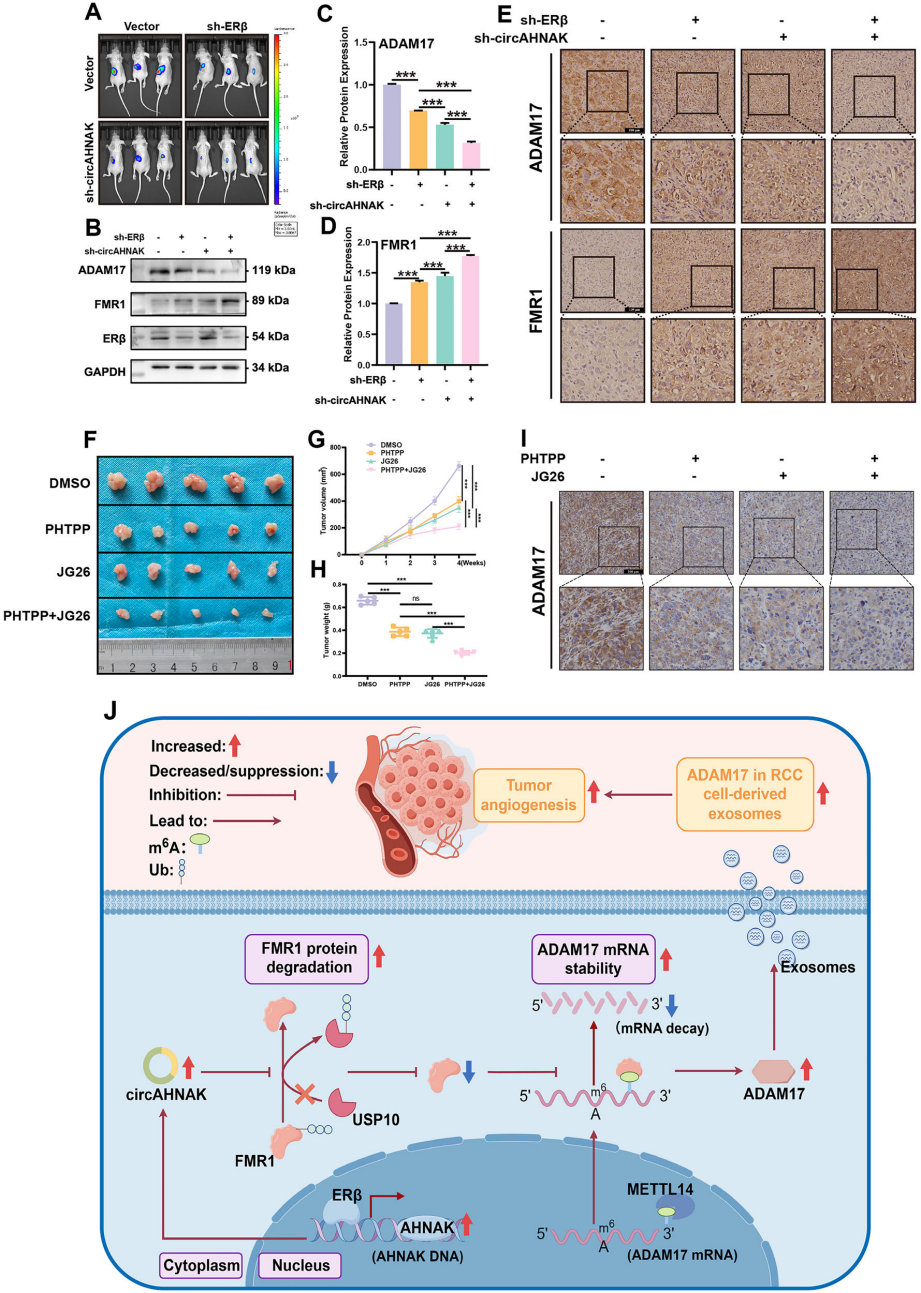
Blocking the ERβ/circAHNAK/FMR1/ADAM17 pathway curbs ccRCC growth. A) Bioluminescent imaging of orthotopic xenograft tumors derived from 786‐O‐Luc cells with stable knockdown of ERβ, circAHNAK, or both, showing significant tumor suppression in all knockdown groups, with the strongest effect observed in the dual‐knockdown group. B–D) Western blot analysis of xenograft tumor tissues demonstrated increased FMR1 and decreased ADAM17 expression upon ERβ or circAHNAK knockdown, with the most pronounced changes in the dual‐knockdown group. E) Representative IHC staining of FMR1 and ADAM17 in tumor sections corroborates Western blot findings. F) Tumor growth curves in syngeneic Renca subcutaneous models treated with vehicle, PHTPP(3 mg/kg), JG26(3 mg/kg), or combination, showing synergistic suppression of tumor growth with dual treatment. G,H) Tumor volume and weight measurements at endpoint. I) IHC analysis confirmed reduced ADAM17 expression in tumors from PHTPP‐ and JG26‐treated mice. J) Schematic illustration of the proposed model, ERβ upregulates circAHNAK, which binds and destabilizes FMR1 by interfering with USP10‐mediated deubiquitination, leading to increased ADAM17 stability and enhanced angiogenesis and tumor progression in ccRCC. Targeted inhibition of this axis effectively suppresses tumor growth. Data represent mean ± SEM. ^*^ *P* < 0.05, ^**^ *P* < 0.01, ^***^ *P* < 0.001 vs control. Scale bar = 100 µm.

To evaluate the in vivo therapeutic value of the ERβ/circAHNAK/FMR1/ADAM17 axis, orthotopic xenograft models were established by implanting 786‐O‐Luc cells with stable knockdown of ERβ, circAHNAK, or both into the renal subcapsular space of nude mice. Bioluminescent imaging (IVIS) demonstrated that silencing either ERβ or circAHNAK significantly impeded tumor growth, with the most pronounced suppression observed in the dual‐knockdown group (**Figure** **10**A). Western blotting (Figure 10B–D) and IHC (Figure 10E) confirmed that ERβ or circAHNAK depletion increased FMR1 and decreased ADAM17 levels, with dual knockdown showing the most pronounced effect.

To assess the therapeutic potential of pharmacologically targeting this pathway, a syngeneic Renca cell subcutaneous model was employed in immunocompetent Balb/c mice. Treatment with the selective ERβ antagonist PHTPP or the ADAM17 inhibitor JG26 significantly inhibited tumor growth, with combined treatment showing synergistic efficacy (Figure 10F). Tumor volume and weight measurements further substantiated these findings (Figure 10G,H). Mechanistically, JG26 treatment alone suppressed ADAM17 protein expression, and this suppression was further potentiated by co‐administration of PHTPP (Figure 10I).

Taken together, these findings highlight the critical role of the ERβ/circAHNAK/FMR1/ADAM17 axis in ccRCC progression and progression, suggesting that targeted inhibition of this pathway may represent a promising therapeutic strategy for ccRCC (Figure 10J).

## 3 Discussion

ERβ, a member of the nuclear receptor superfamily, regulates gene transcription upon binding to estrogenic ligands such as 17β‐estradiol. Ligand‐bound ERβ forms dimers and recruits coactivators or corepressors to EREs within target gene promoters, thereby modulating transcriptional activity.^[^ ^40^, ^41^, ^42^ ^]^ The biological role of ERβ is highly tissue‐specific, in breast cancer, ERβ often antagonizes ERα, suppressing cell proliferation.^[^ ^43^, ^44^ ^]^ Conversely, in various solid tumors, including lung, bladder, and ccRCC, ERβ is frequently overexpressed and facilitates tumor progression by regulating noncoding RNAs, promoting angiogenesis, and enhancing invasion and therapeutic resistance.^[^ ^9^, ^45^, ^46^ ^]^ In ccRCC, ERβ directly binds an ERE within the AHNAK promoter, transcriptionally upregulating circAHNAK. circAHNAK subsequently interacts with FMR1, impeding its deubiquitination by USP10 and thereby accelerating FMR1 degradation. The resulting loss of FMR1 disrupts m⁶A‐dependent recognition and decay of ADAM17 mRNA, leading to ADAM17 accumulation and enhanced tumor angiogenesis. This delineates a novel mechanism whereby ERβ regulates noncoding RNAs and orchestrates post‐transcriptional and post‐translational modifications to promote tumor angiogenesis.

CircRNAs are covalently closed, highly stable RNA molecules produced by back‐splicing of pre‐mRNAs.^[^ ^12^ ^]^ Beyond their established roles as microRNA sponges, circRNAs modulate gene expression through interactions with RBPs, thereby influencing RBP stability, localization, and activity.^[^ ^13^ ^]^ circAHNAK was markedly upregulated in ccRCC tissues and cell lines, correlating positively with microvessel density in patient samples. Various experiments validate circAHNAK as a pivotal downstream regulator of ERβ‐mediated ccRCC angiogenesis.

FMR1 is an evolutionarily conserved RNA‐binding protein that contains multiple RNA‐binding modules, including two KH domains and a C‐terminal RGG box, which mediate sequence‐ and structure‐specific interactions with target RNAs.^[^ ^47^ ^]^ Functionally, FMR1 regulates mRNA transport, stability, and translation, often assembling into ribonucleoprotein granules to modulate target mRNA fate at the post‐transcriptional level.^[^ ^48^ ^]^ Recent studies implicate FMR1 as an important m⁶A reader predominantly characterized in the context of neurodevelopment.^[^ ^25^, ^26^ ^]^ However, its binding specificity and functional spectrum in cancer remain poorly defined. In our study, ADAM17 emerged as a key m⁶A‐modified target of FMR1, representing only a subset of its potential regulatory repertoire. Our future research will integrate RNA–protein interactome mapping (e.g., CLIP‐seq or RIP‐seq) with complementary proteomic and transcriptome analyses to comprehensively delineate FMR1‐associated networks in RCC. These investigations are expected to clarify the role of FMR1 in m⁶A‐mediated RNA regulation and potentially uncover additional downstream effectors within the ERβ/circAHNAK axis.

Deubiquitination, catalyzed by DUBs, removes polyubiquitin chains from substrates, reversing ubiquitination and thereby stabilizing or modulating protein function.^[^ ^18^ ^]^ USP10, a cysteine protease in the USP family, recognizes diverse ubiquitin chain linkages via its catalytic domain.^[^ ^19^, ^20^ ^]^ USP10 acts as the principal DUB for FMR1, specifically targeting the C‐terminal K593 residue to remove polyubiquitin chains and prevent proteasomal degradation. CircAHNAK competes with USP10 for FMR1 binding, thereby inhibiting deubiquitination and promoting rapid ubiquitination and degradation of FMR1. Consequently, FMR1 loss impairs binding to m⁶A‐marked ADAM17 mRNA and recruitment of decay complexes, stabilizing ADAM17 transcripts, increasing protein levels, and enhancing angiogenesis in ccRCC cells. Notably, although the K593R mutation of FMR1 partially rescued its degradation, this restoration was incomplete. This observation suggests that K593 may not be the sole ubiquitination site governing FMR1 stability. Other lysine residues may contribute to its polyubiquitination, or circAHNAK indirectly modulates alternative degradation pathways independent of K593. Moreover, other deubiquitinating enzymes, beyond USP10, could act redundantly or cooperatively to regulate FMR1 turnover under specific cellular conditions. In future research, we plan to employ systematic mutagenesis of multiple lysine residues, together with global ubiquitination mapping, to fully elucidate these mechanisms. These investigations will refine our understanding of how circAHNAK–FMR1 interactions regulate post‐translational stability and downstream m⁶A‐mediated RNA regulation in RCC.

m⁶A methylation critically regulates RNA metabolism, including stability, splicing, translation, and localization, and is increasingly recognized as a key modulator of tumor angiogenesis.^[^ ^8^, ^49^ ^]^ The METTL3/METTL14/WTAP methyltransferase complex deposits m⁶A marks, whereas FTO and ALKBH5 mediate demethylation, and various “reader” proteins such as YTHDF, IGF2BP, and HNRNP determine RNA fate.^[^ ^21^, ^22^ ^]^ We show that METTL14 catalyzes m⁶A deposition in the 3′‐UTR of ADAM17 mRNA, generating binding sites for FMR1 that facilitate the recruitment of RNA decay machinery. Interestingly, METTL14 knockdown results in a more pronounced increase in ADAM17 expression than METTL3 knockdown. This may be attributed to METTL14's dual role as a scaffold within the writer complex and as a direct RNA‐binding protein via its C‐terminal RGG repeats, which influence site specificity.^[^ ^50^ ^]^ Prior studies have shown that deletion of the RGG domain diminishes METTL14's RNA binding affinity and compromises m⁶A deposition efficiency and fidelity,^[^ ^51^ ^]^ while removal of the C‐terminal RGG repeats abrogates the catalytic activity of the METTL3/METTL14 heterodimer.^[^ ^52^ ^]^ These findings explain METTL14's predominant role in ADAM17 mRNA modification and turnover.

ADAM17 (TACE) is a membrane‐bound metalloprotease that promotes angiogenesis via multiple mechanisms within the tumor microenvironment. It cleaves membrane‐bound precursors of pro‐angiogenic factors such as VEGF family members, releasing active ligands that rapidly activate EGFR/ERK signaling pathways in endothelial cells, thereby promoting proliferation and migration.^[^ ^29^, ^53^ ^]^ Additionally, ADAM17 regulates matrix metalloproteinase activity to remodel the extracellular matrix and facilitate neovascularization.^[^ ^54^ ^]^ Loss of ADAM17 in endothelial cells impairs their physiological function.^[^ ^28^, ^55^ ^]^ Recent evidence indicates that tumor cells package ADAM17 into exosomes and release them. When endothelial cells take up these exosomes, ADAM17 not only continues to cleave pro‐angiogenic ligands but also increases vascular permeability and induces leakage, thereby establishing a pre‐metastatic niche.^[^ ^39^ ^]^ Collectively, ADAM17 exerts a dual role in ccRCC angiogenesis, it not only facilitates pro‐angiogenic factor release at the tumor cell surface but is also delivered to endothelial cells via exosomes, amplifying pro‐angiogenic signaling. This multifaceted mechanism may underlie tumor resistance to conventional anti‐VEGF therapies.

Although the ERβ/circAHNAK/FMR1/ADAM17 signaling axis has been extensively validated through in vitro and in vivo experiments, several limitations remain. Clinical studies correlating pathway component expression with patient prognosis are necessary to confirm clinical relevance. The specific endothelial receptors and downstream cascades activated by exosomal ADAM17 also remain to be identified, and the potential effects of this axis on ccRCC invasion, metastasis, and immune evasion require further exploration. Moreover, these pathway components may engage in crosstalk with multiple signaling pathways, potentially contributing to ccRCC progression. For instance, ERβ has been reported to interact with the PI3K–AKT and MAPK pathways to regulate transcriptional programs^[^ ^56^ ^]^; FMR1 may modulate the RNA fate of critical effectors in Wnt and EGFR signaling^[^ ^57^, ^58^ ^]^; and ADAM17 has been implicated in activating Notch and TGF‐β signaling.^[^ ^59^, ^60^ ^]^ Future studies are warranted to systematically investigate these potential crosstalks.

In summary, these findings demonstrate that ERβ binds to ERE in the AHNAK promoter to drive circAHNAK biogenesis. CircAHNAK competitively inhibits USP10‐mediated deubiquitination of FMR1, promoting its K593‐linked polyubiquitination and proteasomal degradation. Loss of FMR1 compromises the recognition of m⁶A‐modified ADAM17 transcripts, allowing ADAM17 mRNA to evade METTL14‐dependent degradation and increased ADAM17 protein accumulation. Secreted via exosomes, elevated ADAM17 enhances endothelial cell angiogenic activity within the tumor microenvironment. This study is the first to integrate transcriptional regulation with post‐transcriptional and post‐translational modifications in driving ccRCC angiogenesis. These findings deepen the mechanistic understanding of ccRCC angiogenesis and underscore the ERβ/circAHNAK/FMR1/ADAM17 axis as a promising therapeutic target.

## 4 Experimental Section

### Patient Specimens and Public Patient Cohorts

This study collected 38 paired ccRCC tumor tissues and adjacent normal tissues from patients treated at the Second Hospital of Hebei Medical University between June 2021 and June 2023 for subsequent experimental analysis. All participants provided written informed consent, and the study protocol was approved by the Ethics Committee of the Second Hospital of Hebei Medical University (approval No. 2020‐R373).

CircRNA sequencing data for ccRCC were obtained from the GEO database (accession numbers GSE100186 and GSE137836(https://www.ncbi.nlm.nih.gov/geo/).^[^ ^61^, ^62^ ^]^ In addition, m⁶A RNA methylation sequencing (MeRIP‐seq) data for ccRCC were obtained from GEO under accession number GSE262500.^[^ ^63^ ^]^ Proteomic data and clinical parameters were acquired from the Clinical Proteomic Tumor Analysis Consortium (CPTAC) database (https://cptac‐data‐portal.georgetown.edu/).

### Cell Lines and Transfection

Human umbilical vein endothelial cells (HUVECs; RRID, CVCL_2959) were purchased from the Cell Bank of the Chinese Academy of Sciences (Beijing, China) and cultured in Dulbecco's Modified Eagle Medium (DMEM; Gibco, USA) supplemented with 10% fetal bovine serum (FBS; Gibco, USA). Human renal carcinoma cell lines 786‐O (RRID, CVCL_1051), A498 (RRID, CVCL_1056), OSRC‐2 (RRID, CVCL_1626), ACHN (RRID, CVCL_1067), and 769‐P (RRID, CVCL_1050), and the mouse renal carcinoma cell line Renca (RRID, CVCL_2174) were obtained from Procell (Wuhan, China). 786‐O, OS‐RC‐2, ACHN, 769‐P, and Renca cells were maintained in RPMI‐1640 medium (Gibco, USA) with 10% FBS, while A498 cells were cultured in Minimum Essential Medium (MEM; Gibco, USA) with 10% FBS. HEK293T cells (RRID, CVCL_0063) were purchased from Procell and cultured in DMEM supplemented with 10% FBS for lentiviral packaging. All cell lines were authenticated by short tandem repeat (STR) profiling and tested negative for mycoplasma contamination before use. Cells were cultured at 37 °C in a humidified 5% CO_2_ incubator. Transfection was performed using Lipofectamine 2000 (Thermo Fisher, USA) according to the manufacturer's protocol. All siRNAs (gene‐specific and control), plasmids (pcDNA3.1‐oeUSP10, pcDNA3.1‐oeMETTL14, Flag‐FMR1‐N, Flag‐FMR1‐M, Flag‐FMR1‐C, Flag‐FMR1‐ΔC), and control vectors were purchased from GenePharma (Shanghai, China).

### Lentiviral Plasmid Construction

Vectors were constructed as previously described. HEK‐293T cells were co‐transfected with pLKO.1‐shERβ, pWPI‐oeERβ, pLKO.1‐shcircAHNAK, pLCDH‐ciR‐oecircAHNAK, pLKO.1‐shFMR1, pWPI‐oeFMR1, pLKO.1‐ADAM17, pWPI‐ADAM17, psPAX2 packaging plasmid, and pMD2.G envelope plasmid using standard calcium phosphate transfection. Lentiviral particles were amplified, harvested from cell culture supernatants, concentrated by density gradient centrifugation, and stored at −80 °C.

### Quantitative Real‐Time PCR (qRT‐PCR)

Total RNA was isolated using the RNA extraction kit (SEVEN, Beijing, China), and subcellular RNA fractions were obtained using the RNA Subcellular Isolation Kit (Active Motif, USA). RNA concentration was measured using a NanoDrop 2000 spectrophotometer (Thermo Fisher, USA). First‐strand cDNA was synthesized using the M‐MLV First Strand Kit (Thermo Fisher, USA). qRT‐PCR was performed on an ABI 7500 FAST system (Applied Biosystems, USA) using Platinum SYBR Green qPCR SuperMix‐UDG (Thermo Fisher, USA). GAPDH and U6 served as reference genes for cytoplasmic and nuclear RNA, respectively. Relative expression was calculated using the 2^−ΔΔ^Ct method. All primer sequences are listed in Table S1 (Supporting Information).

### Western Blot Analysis

Total protein was extracted using RIPA buffer containing a protease inhibitor cocktail as previously described.^[^ ^64^, ^65^ ^]^ Protein concentrations were quantified, and equal amounts were separated by SDS‐PAGE before transferring to polyvinylidene difluoride (PVDF) membranes (Millipore, USA). After blocking with 5% skim milk for 2 h, membranes were incubated with primary antibodies, ERβ (1, 2000, GTX70174, GeneTex, USA), ADAM17 (1, 8000,29948‐1‐AP, Proteintech, China), FMR1(1, 2000,13755‐1‐AP, Proteintech, China), USP10(1, 1000,19374‐1‐AP, Proteintech, China), Ub(1, 10 000,80992‐1‐RR, Proteintech, China), FLAG(1, 20 000, 20543‐1‐AP, Proteintech, China), CD9(1, 30 000, 20597‐1‐AP, Proteintech, China), HSP70 (1, 10 000, 10995‐1‐AP, Proteintech, China), TSG101 (1, 8000, 28283‐1‐AP, Proteintech, China), Calnexin (1, 10 000, 10427‐2‐AP, Proteintech, China), and GAPDH (1, 50 000, 60004‐1‐IG, Proteintech, China). After incubation with HRP‐conjugated secondary antibodies (1, 10 000, RGAR001/RGAM001, Proteintech, China), protein bands were visualized using chemiluminescent HRP substrate (Millipore, USA) and detected with the Fusion FX imaging system (Vilber Lourmat, France). Uncropped blots are provided in Figure S9 (Supporting Information), including results from three independent biological replicates.

### In Vitro Angiogenesis Functional Assays

To evaluate the effects of ccRCC cells on endothelial function, HUVECs were indirectly co‐cultured with ccRCC cells using Transwell inserts (0.4 µm pore size; Corning) for 48 h. HUVECs were subsequently subjected to tube formation, Transwell migration, wound healing, colony formation, EdU incorporation, and CCK‐8 viability assays.

Tube Formation Assay, HUVECs (2 × 10^^3^ cells/well) were seeded in Matrigel‐coated 96‐well plates and incubated for 4–6 h at 37 °C. Tube‐like structures were imaged using an inverted microscope, and total tube length was quantified with ImageJ (*N* = 3).

Transwell Migration Assay, HUVECs (3 × 10^^3^ cells/well) were seeded in the upper chambers of 24‐well Transwell inserts (8 µm pore size; NEST, China) in serum‐free medium. The lower chambers contained medium with 10% FBS. After 24 h, migrated cells were fixed, stained with 0.1% crystal violet, and counted in ≥3 random fields (*N* = 3).

Wound Healing Assay, HUVECs were grown in 6‐well plates to ≈90% confluency and scratched with a 200 µL pipette tip. Detached cells were removed with PBS washes, and wound closure was recorded at 0 and 24 h under an inverted microscope. Residual wound area was quantified using ImageJ in ≥3 randomly selected fields.

Colony Formation Assay, HUVECs (500 cells/well) were seeded in 6‐well plates and cultured for 10–14 days with medium refreshed every 3–4 days. Colonies were fixed with 4% paraformaldehyde, stained with 0.1% crystal violet, washed, and air‐dried. Colonies containing >50 cells were counted under a light microscope (*N* = 3).

EdU Incorporation Assay, Cell proliferation was measured using the EdU kit (Beyotime, China). HUVECs were incubated with 10 µM EdU for 2 h at 37 °C, fixed with 4% paraformaldehyde, permeabilized with 0.5% Triton X‐100, and subjected to the Click reaction. Nuclei were counterstained with DAPI, and EdU‐positive cells were quantified from at least five random fields under a fluorescence microscope (*N* = 3).

CCK‐8 Viability Assay, HUVEC viability was determined using the CCK‐8 kit (SEVEN, China). Cells were seeded into 96‐well plates at the indicated density. At specified time points, 10 µL of CCK‐8 solution was added to each well containing 100 µL medium and incubated for 1–2 h at 37 °C. Absorbance at 450 nm was measured with a microplate reader. Each condition was tested in triplicate and repeated independently three times.

### RNase R Treatment

Total RNA (2 µg) was incubated with RNase R (5 U/µg; Beyotime, China) at 37 °C for 15 min, followed by qPCR analysis.

### Stability Assays

To evaluate RNA and protein stability, 786‐O and A498 cells were treated with transcriptional or translational inhibitors. For RNA stability analysis, cells were seeded in 6‐well plates and treated with 2 µg /mL actinomycin D (MCE, USA). Total RNA was extracted at the indicated time points, and the expression levels of circAHNAK, AHNAK, and ADAM17 were quantified by qPCR.

For protein stability analysis, cells were cultured in T25 flasks and treated with 100 µg /mL cycloheximide (CHX; MCE, USA). At the indicated time intervals, cells were harvested and subjected to Western blotting to assess FMR1 protein levels.

### RNA Fluorescence In Situ Hybridization (RNA‐FISH)

RNA‐FISH was performed using the Fluorescent In Situ Hybridization Kit (GenePharma, China) according to the manufacturer's instructions. Briefly, 5 × 10^3^ renal carcinoma cells were seeded on coverslips in 24‐well plates. After fixation and permeabilization, cells were hybridized with Cy3‐labeled circAHNAK probes overnight at 37 °C. After stringent washing, nuclei were counterstained with DAPI. Images were acquired using a fluorescence microscope (Leica Microsystems, Switzerland).

### Immunohistochemistry (IHC) and Immunofluorescence (IF)

Formalin‐fixed, paraffin‐embedded tissue sections (5 µm) were deparaffinized and rehydrated. For IHC, after antigen retrieval and endogenous peroxidase blocking, sections were incubated with primary antibodies overnight at 4 °C, followed by HRP‐conjugated secondary antibodies and DAB development (ZSGB‐BIO, China). For IF, cells or tissue sections were permeabilized with 0.1% Triton X‐100, blocked with goat serum, and incubated with primary antibodies overnight at 4 °C. After incubation with fluorophore‐conjugated secondary antibodies, nuclei were stained with DAPI. Images were captured using a Leica microscope (Leica Microsystems, Switzerland).

### Luciferase Reporter Assay

The AHNAK promoter sequence and ADAM17 3′‐UTR predicted m⁶A modification sites were cloned into PGL3 AND pSI‐CHECK2 vector (Promega, USA). Site‐directed mutagenesis was performed using the QuikChange Lightning Site‐Directed Mutagenesis Kit (Agilent Technologies, USA). All plasmids were verified by Sanger sequencing and purified using the Omega Bio‐tek Plasmid Mini Kit (D6942, USA). For luciferase assays, 786‐O and A498 cells were co‐transfected with, 1) AHNAK promoter‐reporter constructs/mutants with oe/sh‐ERβ or control vectors; 2) ADAM17 3′‐UTR reporter constructs/mutants with oe‐FMR1 or oe‐METTL14 or control vectors. After 24 h, cells were lysed and analyzed using the Dual‐Luciferase Reporter Assay System (Promega, USA). Luciferase activity was measured with a FLUOstar Omega microplate reader (Berthold Technologies, Germany).

### Chromatin Immunoprecipitation (ChIP) Assay

Following standard protocol, cells were formaldehyde‐fixed (4%), sonicated, and immunoprecipitated with specific antibodies (4 °C overnight; IgG control).^[^ ^9^ ^]^ Target regions in the AHNAK promoter were analyzed by qRT‐PCR (Table S1, Supporting Information). Normal rabbit IgG served as the negative control.

### RNA Immunoprecipitation (RIP)

RIP assays were performed using the RIP Kit (BersinBio, China) following the manufacturer's protocol. Briefly, cells were lysed in RIP lysis buffer containing protease and RNase inhibitors. Lysates were incubated with 5 µg of specific antibodies or control IgG overnight at 4 °C, followed by incubation with Protein A/G magnetic beads for 2 h at 4 °C. After washing, RNA‐protein complexes were eluted, and co‐precipitated RNAs were extracted using TRIzol reagent and analyzed by RT‐qPCR.

### RNA Pull‐Down Assay

RNA pull‐down assays were performed using the RNA Pull‐Down Kit (BersinBio, China) according to the manufacturer's instructions. Biotin‐labeled circAHNAK‐specific or negative control probes were synthesized by GenePharma (Shanghai, China). Cell lysates from 786‐O and A498 cells were incubated with streptavidin‐coated magnetic beads conjugated with biotinylated probes for 2 h at room temperature (25 °C). After extensive washing, RNA‐bound proteins were eluted and subjected to Western blot analysis. Both sense and antisense probes were used as controls to ensure specificity.

### Co‐Immunoprecipitation (Co‐IP)

Co‐IP assays were conducted using the Pierce Classic Magnetic IP/Co‐IP Kit (Thermo Scientific, USA). Cells were lysed in IP lysis buffer supplemented with protease inhibitors. Lysates were incubated with specific antibodies or control IgG for 1 h at room temperature, followed by incubation with pre‐washed Protein A/G magnetic beads for an additional hour. After thorough washing, immunoprecipitated proteins were eluted and analyzed by Western blotting. Input controls were loaded to verify protein expression.

### RNA Electrophoretic Mobility Shift Assay (RNA‐EMSA) with Chemiluminescent Detection

Total RNA was extracted using TRIzol reagent and quantified. RNA‐EMSA was performed under RNase‐free conditions. Protein extracts (5 µL) were incubated with yeast tRNA, RNase inhibitor, and anti‐FMR1 antibody (Proteintech, USA) for 10 min at 25 °C, followed by the addition of biotin‐labeled RNA probes for 10 min. Complexes were separated on 5% native PAGE, transferred to nylon membranes, UV‐crosslinked, and detected with HRP‐conjugated streptavidin using enhanced chemiluminescence (ECL).

### m⁶A RNA Immunoprecipitation (MeRIP)‐qPCR

MeRIP assays were performed using the MeRIP Kit (BersinBio, China) in ccRCC cells following the manufacturer's protocol. Total RNA was extracted and then fragmented using an RNA fragmentation buffer. Fragmented RNA was incubated with a m⁶A‐specific antibody or control IgG overnight at 4 °C, followed by capture with Protein A/G magnetic beads. m⁶A‐enriched RNAs were eluted and analyzed by RT‐qPCR using primers targeting ADAM17 m⁶A modification sites (Table S1, Supporting Information).

### m⁶A Quantification

Global m⁶A levels were measured using the m⁶A RNA Methylation Quantification Kit (Abcam) following the manufacturer's instructions. Equal amounts of total RNA were assayed in triplicate, and m⁶A content was normalized to RNA input.

### Exosome Isolation and Characterization

Exosomes were isolated and purified from conditioned medium (CM) of ccRCC cells by differential ultracentrifugation according to previously established protocols. Exosome morphology was examined by transmission electron microscopy (FEI Tecnai G2 Spirit, USA).^[^ ^66^ ^]^ Exosomal markers (CD9, TSG101, HSP70, and Calnexin) were detected by Western blotting using specific antibodies. Size distribution and concentration were analyzed by nanoparticle tracking analysis (NTA) with the ZetaView system (ParticleMetrix, Germany).

### In Vivo Tumor Models

All animal experiments were approved by the Institutional Animal Care and Use Committee (approval No. 2020‐R373) and conducted in accordance with the NIH Guide for the Care and Use of Laboratory Animals. Specific pathogen‐free (SPF) female BALB/c nude mice (4–6 weeks old) were obtained from Vital River Laboratory Animal Technology Co., Ltd. (Beijing, China). For orthotopic models, 5 × 10⁶ 786‐O‐Luc cells transduced with Vector, shERβ, shcircAHNAK, or shERβ + shcircAHNAK (mixed 1, 1 with Matrigel) were implanted beneath the renal capsule. Tumor growth was monitored weekly by bioluminescence imaging using an IVIS Spectrum Imaging System (PerkinElmer, USA), and tumors were harvested on day 28 for further analysis.

For subcutaneous models, Renca cells (5 × 10⁶) were injected into both flanks. Mice were intravenously administered 3 mg/kg JG26 or 3 mg/kg PHTPP via tail vein injection every other day. Tumor volumes were measured weekly using calipers, and tumors were harvested on day 28 for subsequent analysis.

### Structural Modeling and Molecular Docking

The 3D structures of circAHNAK, FMR1, and USP10 were modeled using the computational tools 3dRNA and AlphaFold2.^[^ ^67^, ^68^ ^]^ Molecular docking was subsequently performed with HDOCK under default parameters.^[^ ^69^ ^]^ PyMOL was used for structural visualization and analysis of the docking results.

### Statistical Analysis

Data are presented as mean ± SEM from at least three independent experiments (*n* ≥ 3). Statistical analyses were conducted using IBM SPSS (version 25), GraphPad Prism (version 8.0), and R (version 4.3). Comparisons between the two groups were performed using an unpaired two‐tailed Student's *t*‐test. Survival analysis was conducted using Kaplan‐Meier curves with the log‐rank test. A p‐value less than 0.05 was considered statistically significant.

### Ethics Approval and Consent to Participate

The studies involving human participants were reviewed and approved by the Ethics Committee of the Second Hospital of Hebei Medical University (2020‐R373).

## Conflict of Interest

The authors declare no competing interests.

## Authors' Contribution

C.X. contributed to conceptualization, methodology, and validation. S.Z. and Y. L. were responsible for software development, validation, and visualization. J.L. and Y.C. contributed to writing—both the original draft and review, and editing. Y.C. and H.S. conducted the investigation and formal analysis. S.D. and B.Z. were involved in project administration and investigation. J.L. and X.L. contributed to conceptualization and methodology. B.S., Q.S., and M.Z. were responsible for data curation. J.G. provided supervision and project administration.

## Supporting information



Supporting Information

## Data Availability

The data that support the findings of this study are openly available in GEO database at https://www.ncbi.nlm.nih.gov/geo/, reference number 100186, 137836, 262500. These data were derived from the following resources available in the public domain, Repository: GEO; Accession number: GSE100186, GSE137836, GSE262500; DOI: N/A.
