# Supplementary material for: ERβ/circAHNAK Axis Inhibits USP10–FMR1 Deubiquitination to Prevent m⁶A‐Mediated ADAM17 Decay and Promote Angiogenesis in Clear Cell Renal Cell Carcinoma
Source: Adv Sci (Weinh). 2025 Oct 7;12(48):e09654. doi: 10.1002/advs.202509654 (PMC12752611; doi:10.1002/advs.202509654)
Supplement: Supplementary file 1 — Supporting Information [file ADVS-12-e09654-s001.docx]

**Supplementary Information**


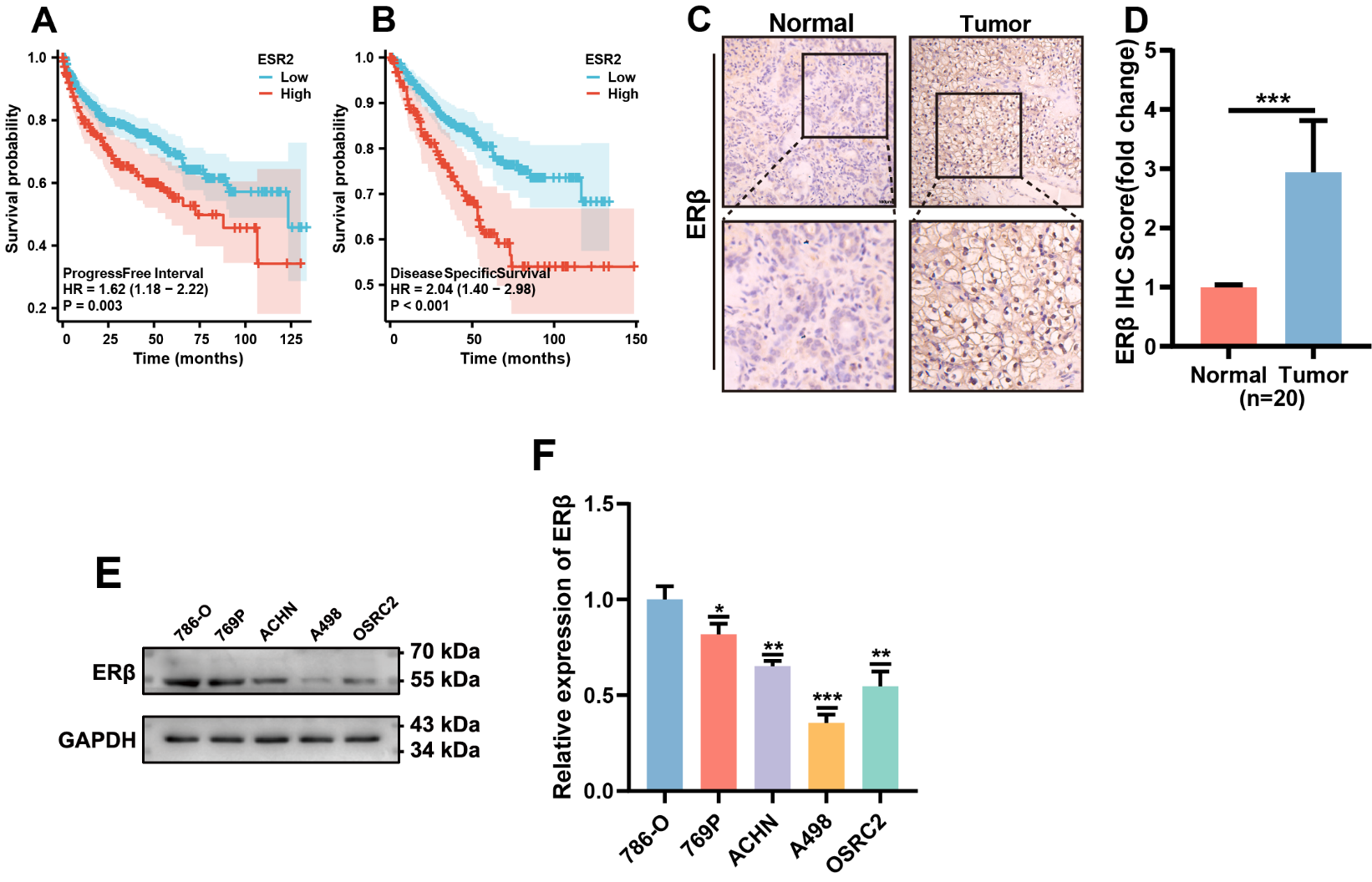


**Supplementary Figure 1. Expression and clinical relevance of ERβ in RCC.**(A, B) Kaplan–Meier survival analyses of disease-specific survival (DSS) and progression-free interval (PFI) in TCGA-KIRC cohort stratified by ERβ expression. (C, D) Representative immunohistochemistry (IHC) images showing differential ERβ expression in RCC tumor tissues compared with matched adjacent normal tissues. (E) Western blot analysis of ERβ protein levels across multiple RCC cell lines, including 786-O, 769P, ACHN, A498, and OSRC-2. Data are presented as mean ± SEM from at least three independent experiments. *P < 0.05, **P < 0.01, ***P < 0.001. Scale bar = 100 μm for IHC images.


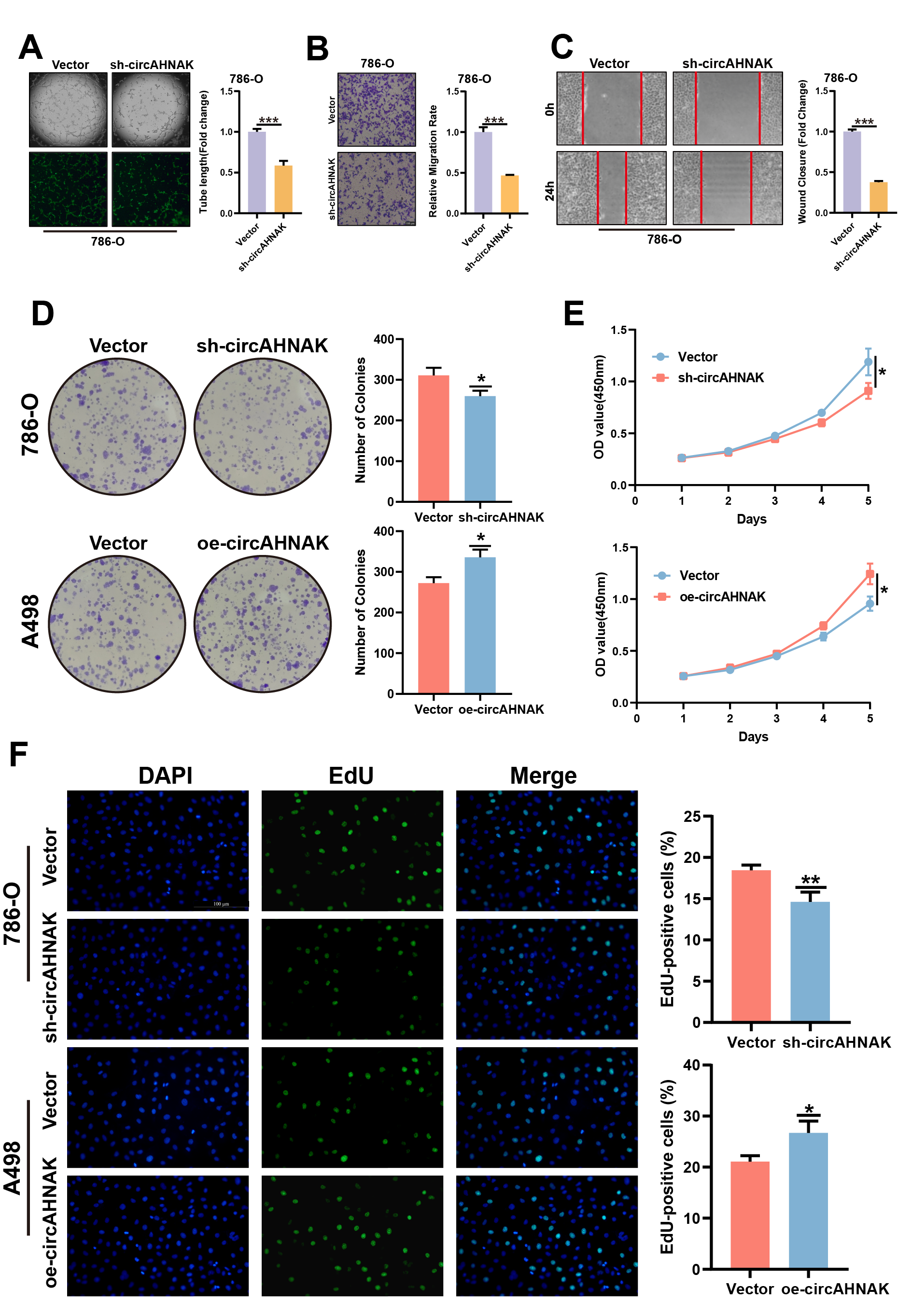


**Supplementary Figure 2. circAHNAK regulates angiogenesis and endothelial cell proliferation in RCC.**(A–C) Effects of circAHNAK knockdown (sh-circAHNAK) on HUVEC angiogenic phenotypes. Tube formation (A), wound healing (B), and transwell migration (C) assays show reduced angiogenic capacity compared with control. (D–F) Effects of circAHNAK knockdown or overexpression on HUVEC proliferation. Colony formation (D), CCK-8 (E), and EdU incorporation (F) assays reveal stepwise changes in proliferation consistent with circAHNAK modulation. Data are mean ± SEM from at least three independent experiments. *P < 0.05, **P < 0.01, ***P < 0.001 vs. control. Scale bar = 100 μm.


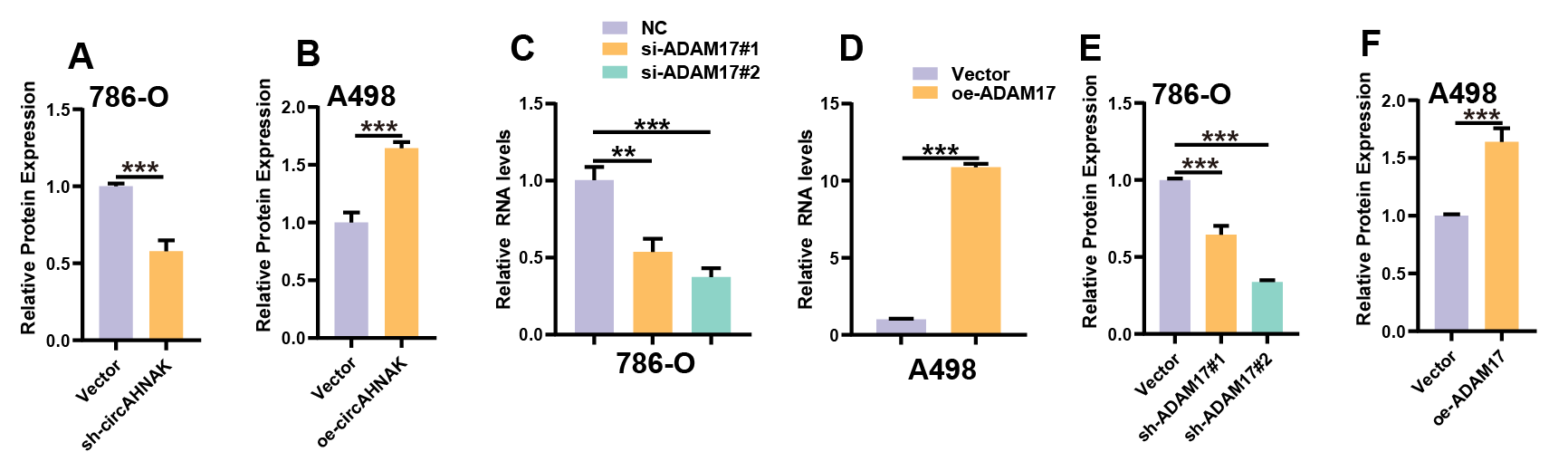


**Supplementary Figure 3. Quantification of circAHNAK- and ADAM17-modulated expression in RCC cells.** (A, B) Quantification of ADAM17 protein levels after circAHNAK knockdown or overexpression. (C, D) qRT-PCR validation of si-ADAM17 and oe-ADAM17 efficiency. (E, F) Quantification of ADAM17 expression after knockdown or overexpression. Data are mean ± SEM from at least three independent experiments. *P < 0.05, **P < 0.01, ***P < 0.001 vs. control.


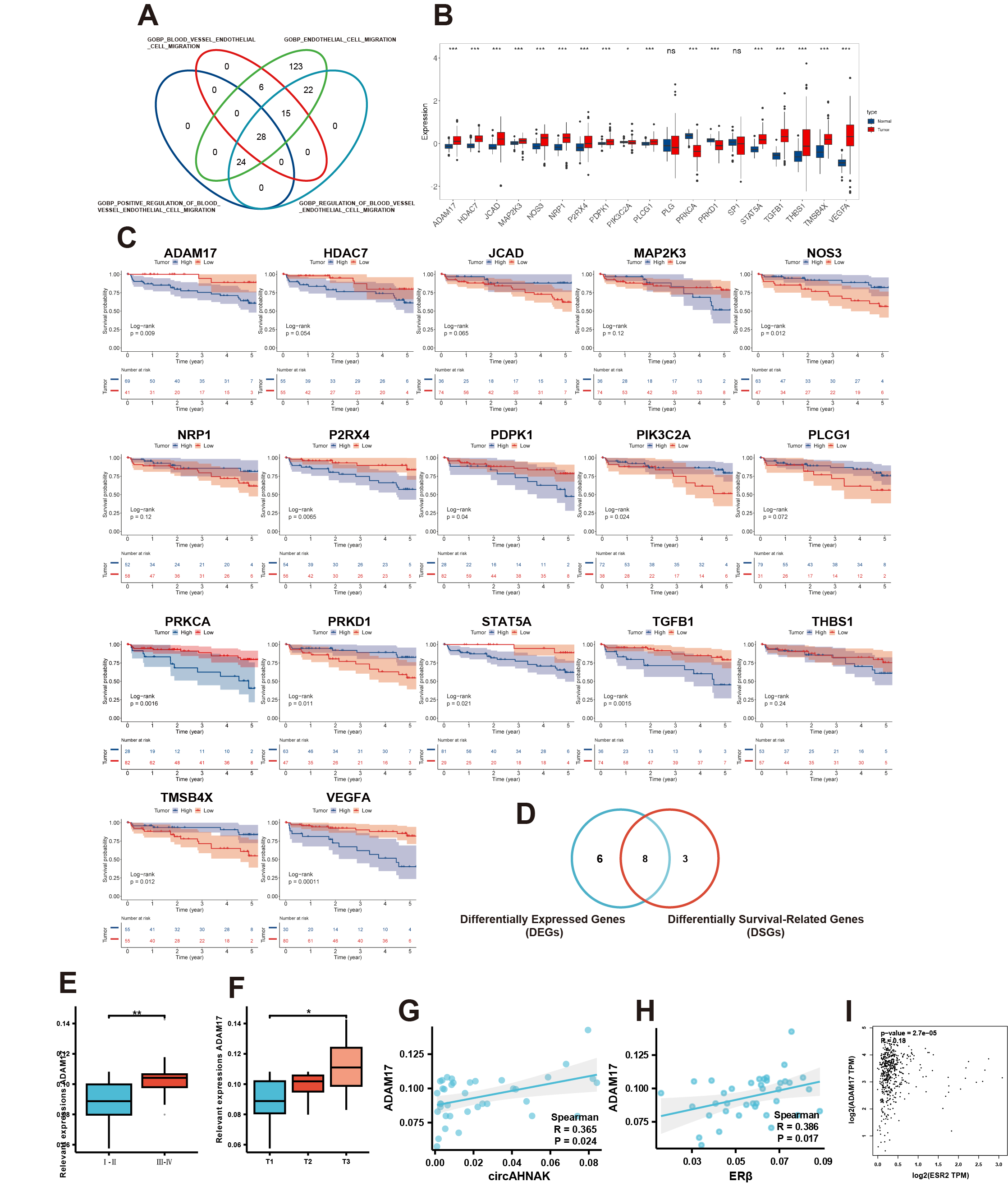


**Supplementary Figure 4. Bioinformatics and clinical validation of angiogenesis-related target genes in ccRCC.** (A) Angiogenesis-related genes were identified from the GO database, and their intersections were visualized by a Venn diagram. (B) Differential expression analysis of intersecting genes was performed using the CPTAC-ccRCC database. (C) Prognostic analysis of differentially expressed genes was performed. (D) The Venn diagram highlights angiogenesis-related genes with both differential expression and prognostic significance in ccRCC. (E, F) qRT-PCR validation in 38 paired RCC patient tissues showed that ADAM17 expression correlates with Fuhrman grade and T stage. (G, H) qRT-PCR validation in the same cohort demonstrated significant positive correlations of ADAM17 with ERβ and circAHNAK expression. (I) Correlation between ADAM17 and ERβ expression in TCGA-KIRC. Data are mean ± SEM from at least three independent experiments. *P < 0.05, **P < 0.01, ***P < 0.001 vs. control.


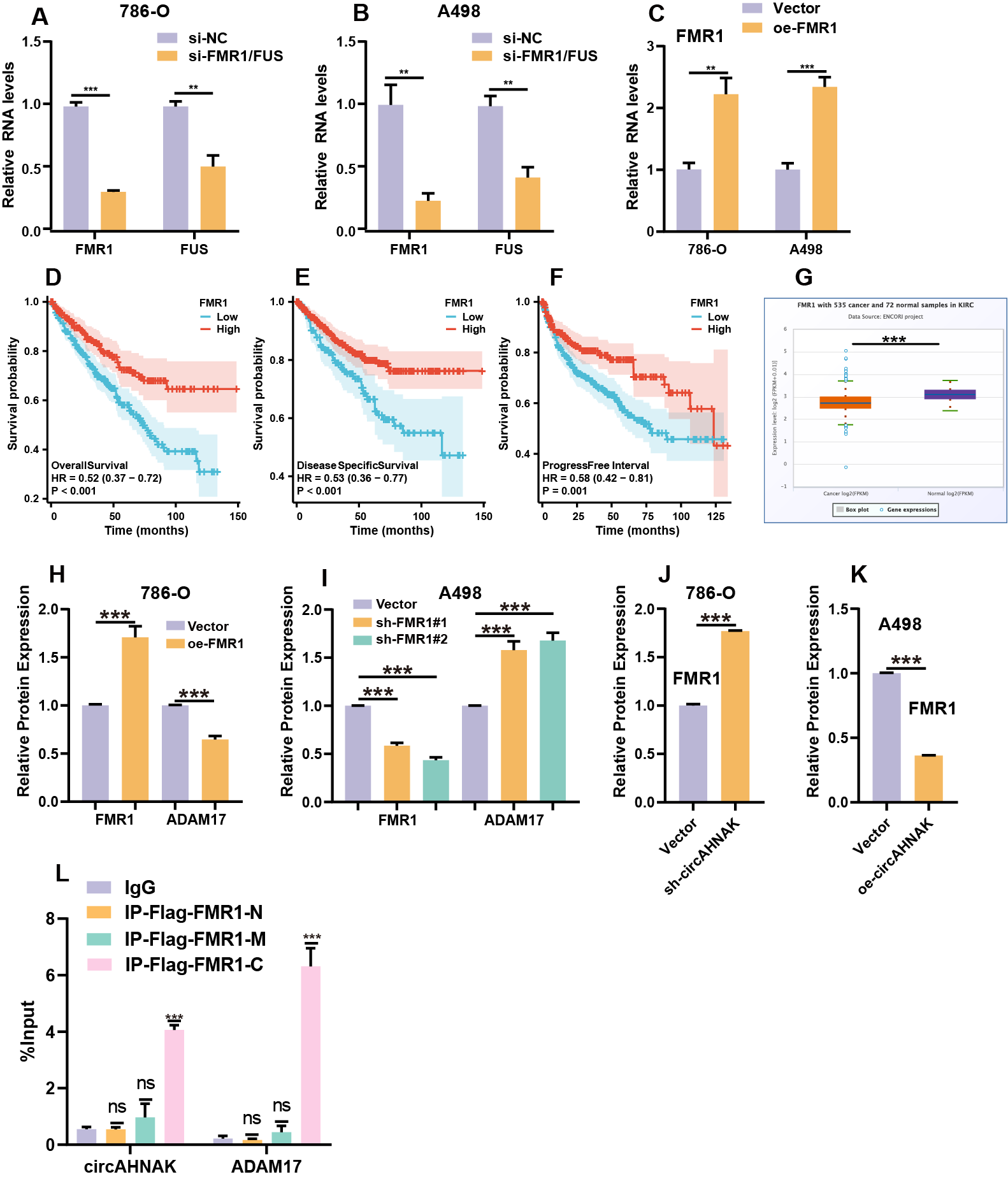


**Supplementary Figure 5. Expression and functional validation of FMR1 in RCC cells.** (A, B) qRT-PCR validation of FMR1 and FUS knockdown efficiency in 786-O and A498 cells. (C) qRT-PCR validation of FMR1 overexpression efficiency in 786-O and A498 cells. (D–F) Differences in overall survival (OS), disease-specific survival (DSS), and progression-free interval (PFI) stratified by FMR1 expression in TCGA-KIRC. (G) Differential expression of FMR1 between ccRCC tumors and adjacent normal tissues in TCGA-KIRC. (H, I) Quantification of FMR1 and ADAM17 protein levels in 786-O and A498 cells after FMR1 knockdown or overexpression. (J, K) Quantification of FMR1 protein levels after circAHNAK knockdown or overexpression. (L) RIP assay using three truncated FMR1 constructs. Data are mean ± SEM from at least three independent experiments. *P < 0.05, **P < 0.01, ***P < 0.001 vs. control.


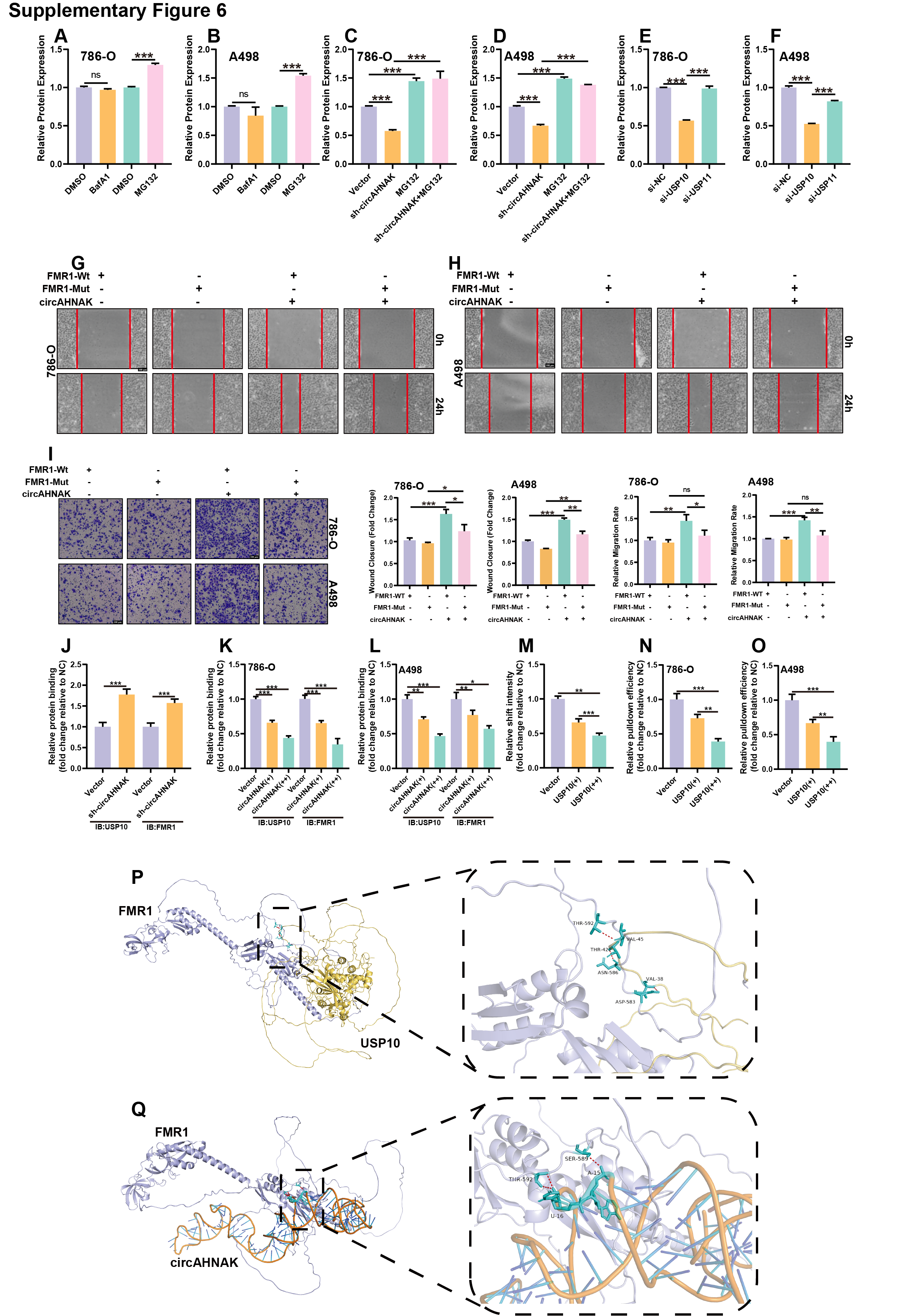


**Supplementary Figure 6. Regulation of FMR1 stability and function by circAHNAK, USP10/USP11, and FMR1 K593R mutant in RCC cells.** (A, B) Quantification of FMR1 protein levels in 786-O and A498 cells after treatment with BafA1 or MG132, as assessed by Western blot. (C, D) Quantification of FMR1 levels in 786-O and A498 cells overexpressing circAHNAK following MG132 treatment. (E, F) Western blot quantification of FMR1 levels in 786-O and A498 cells after knockdown of USP10 or USP11. (G–I) Scratch wound healing and transwell migration assays, along with their quantification, in 786-O and A498 cells transfected with FMR1-WT or FMR1-K593R in circAHNAK rescue experiments. (J-O) Quantification of EMSA, RNA pulldown, and Co-IP results (fold change relative to NC). (P) Docking model of FMR1 (C-terminal region) with USP10, showing key interacting residues Asp-583, Asn-586, and Thr-592. Hydrogen bonds are depicted as dashed lines. (Q) Docking model of FMR1 with circAHNAK, highlighting interactions with Thr-592 and Ser-589. Thr-592 serves as a shared binding site with USP10, suggesting potential competitive binding.Data are mean ± SEM from at least three independent experiments. *P < 0.05, **P < 0.01, ***P < 0.001 vs. control. Scale bar = 100 μm


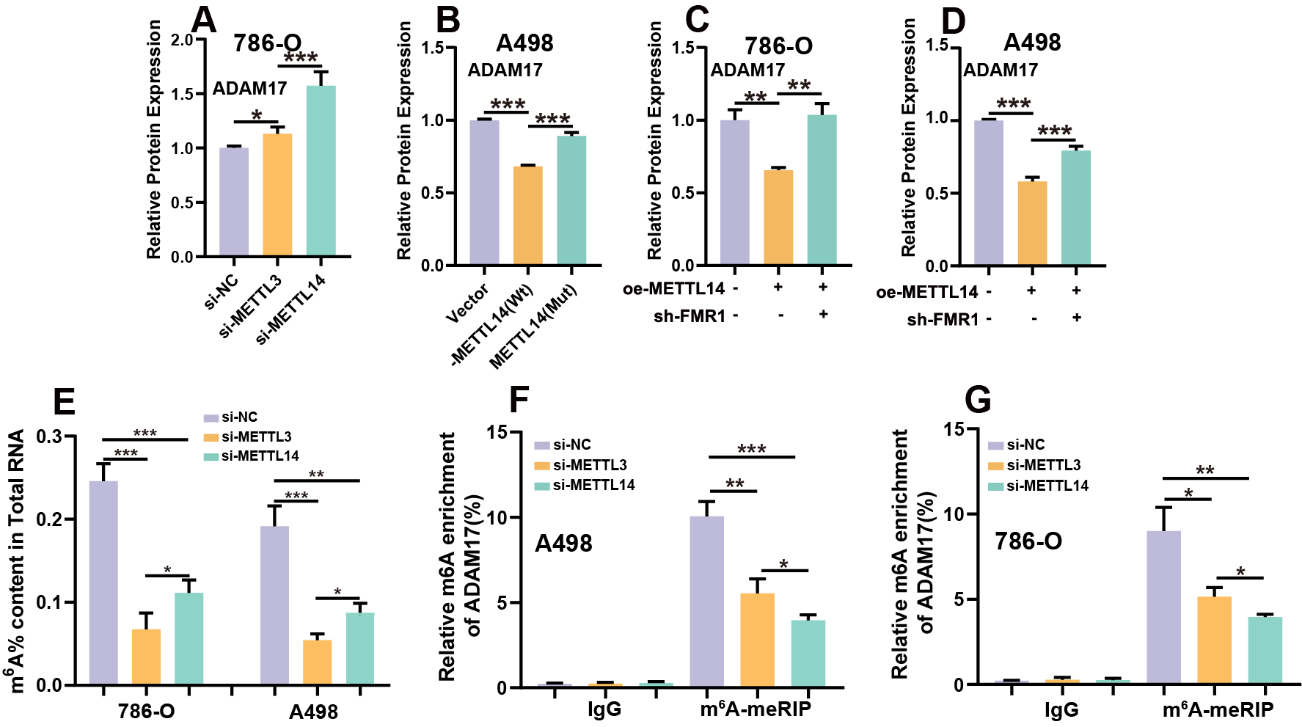


**Supplementary Figure 7. METTL14 regulates ADAM17 expression via m⁶A modification in RCC cells.** (A) Quantification of ADAM17 protein levels in RCC cells following knockdown of METTL3 or METTL14. (B) Quantification of ADAM17 protein levels after transfection with METTL14-WT or METTL14-R298E. (C, D) Rescue experiments showing ADAM17 protein levels in RCC cells co-transfected with METTL14 overexpression and FMR1 knockdown. (E) Global m⁶A levels in RCC cells after METTL3 or METTL14 knockdown, as assessed by m⁶A RNA methylation assay. (F, G) meRIP-qPCR quantification of ADAM17 m⁶A levels in RCC cells following METTL3 or METTL14 knockdown. Data are mean ± SEM from at least three independent experiments. *P < 0.05, **P < 0.01, ***P < 0.001 vs. control.


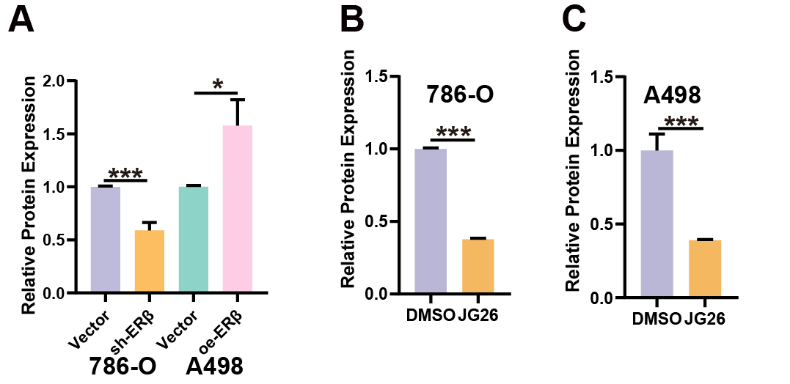


**Supplementary Figure 8. Effects of ERβ modulation and JG26 treatment on exosomal ADAM17 levels.** (A) Quantification of ADAM17 protein levels in exosomes derived from RCC cells after ERβ knockdown or overexpression. (B) Quantification of exosomal ADAM17 protein levels in 786-O and A498 cells following treatment with JG26. Data are mean ± SEM from at least three independent experiments. *P < 0.05, **P < 0.01, ***P < 0.001 vs. control.


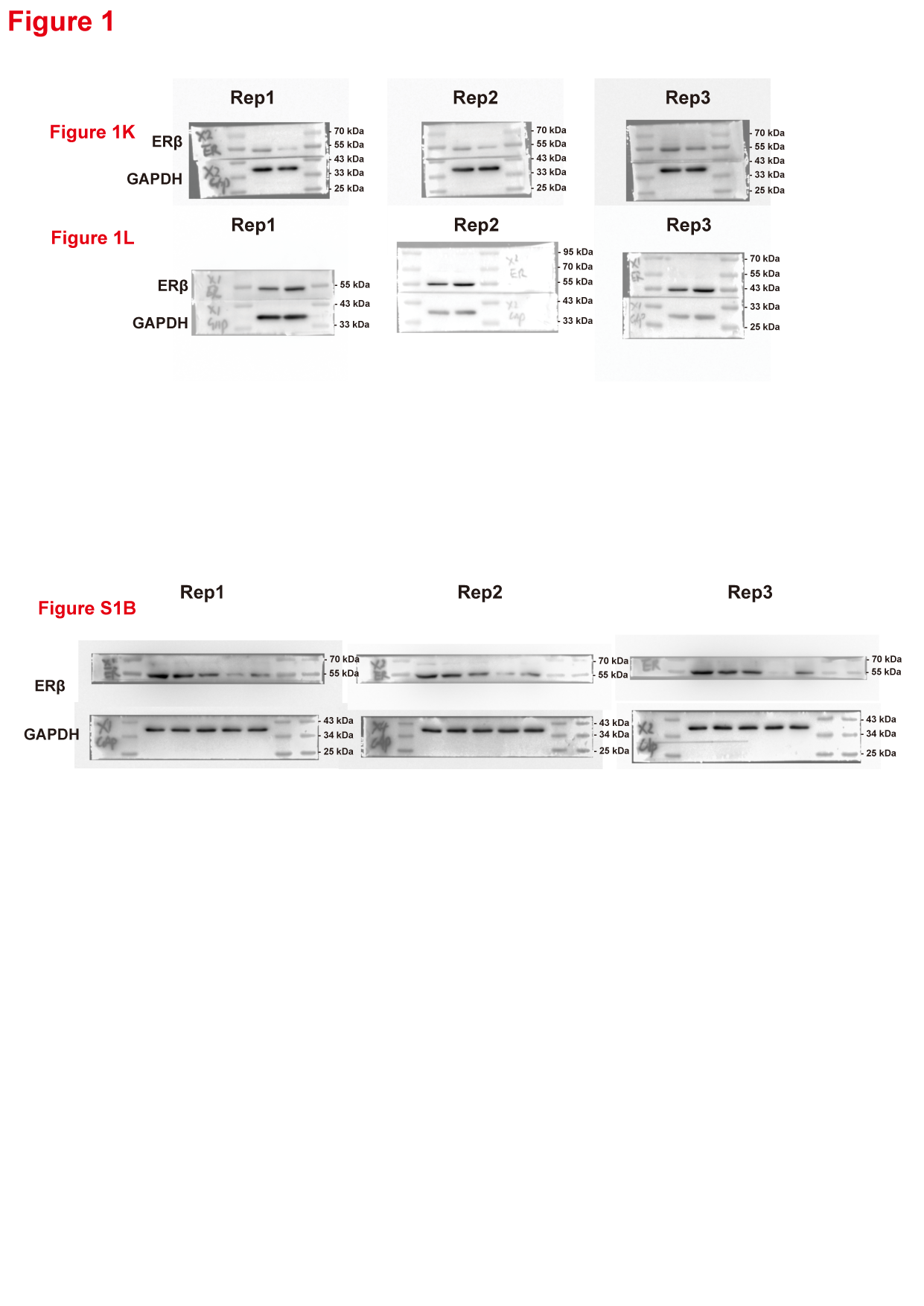


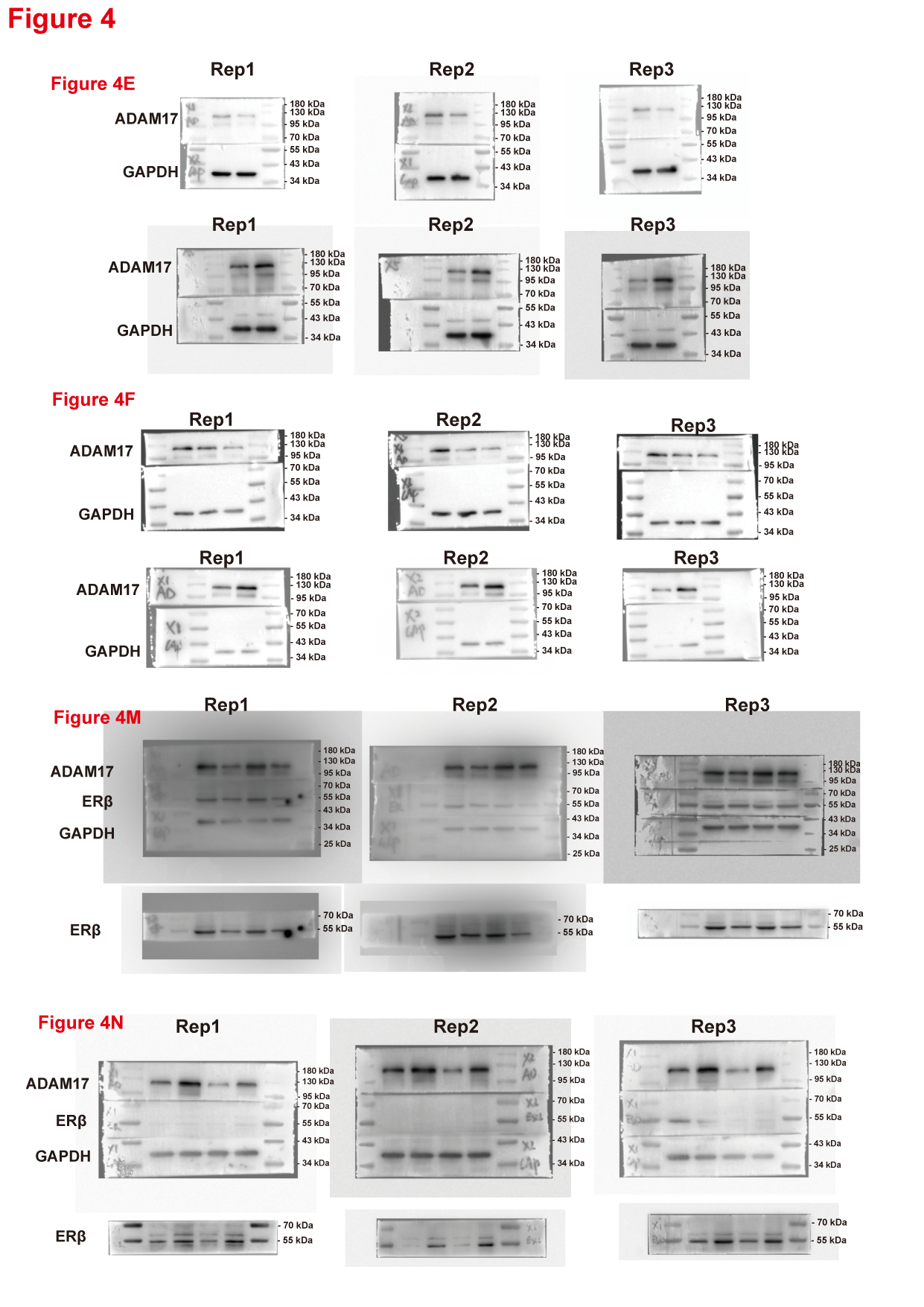


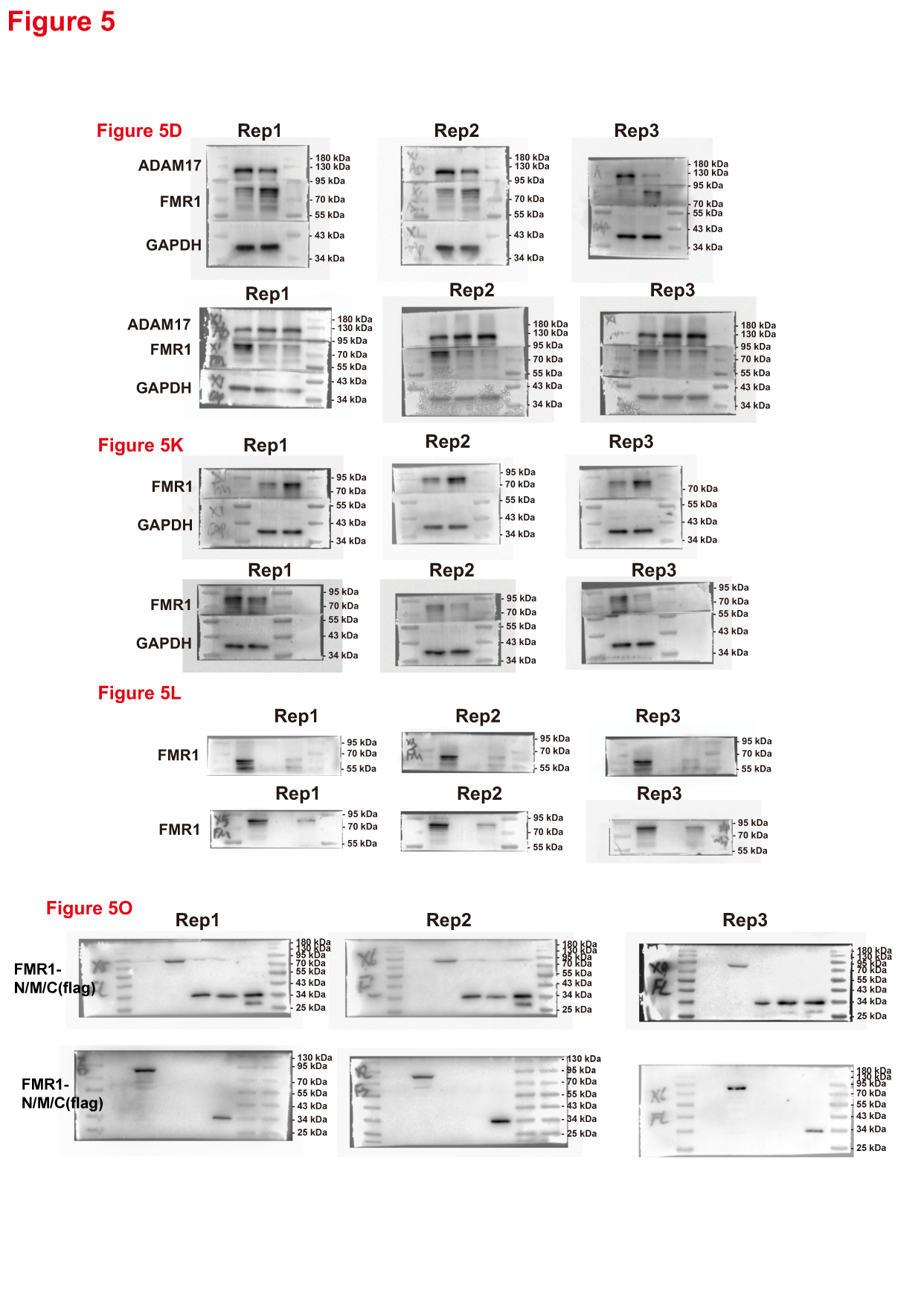


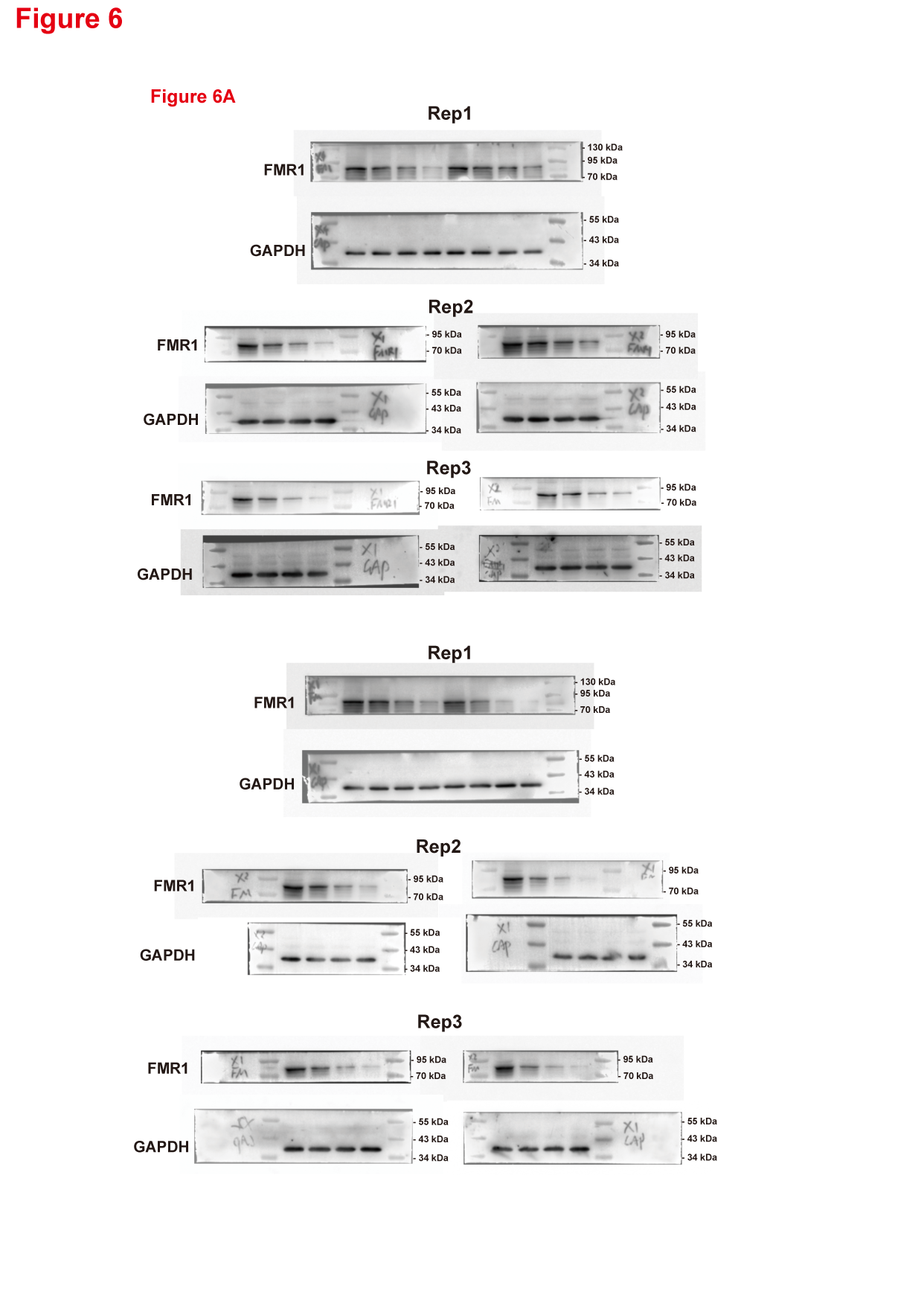


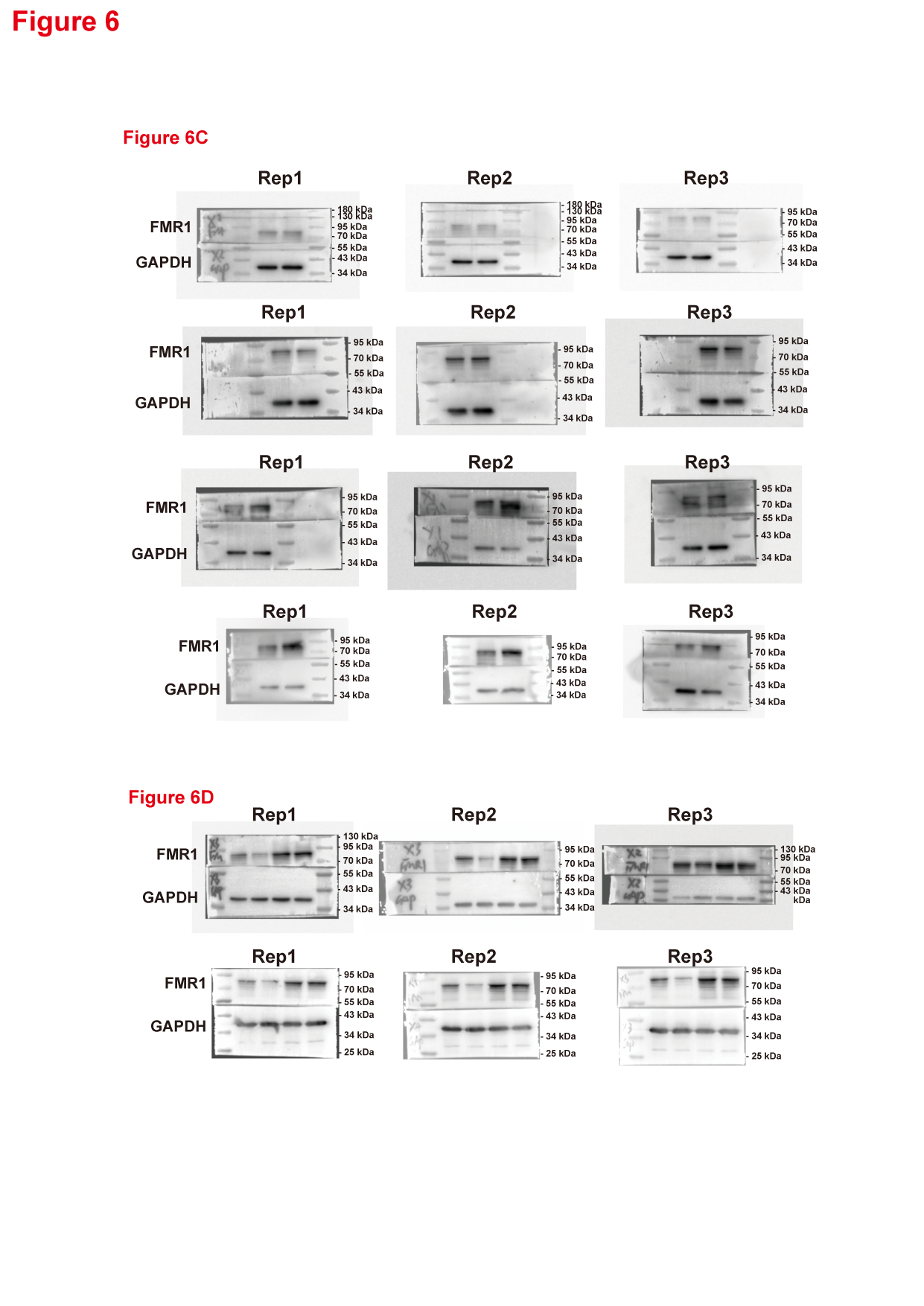


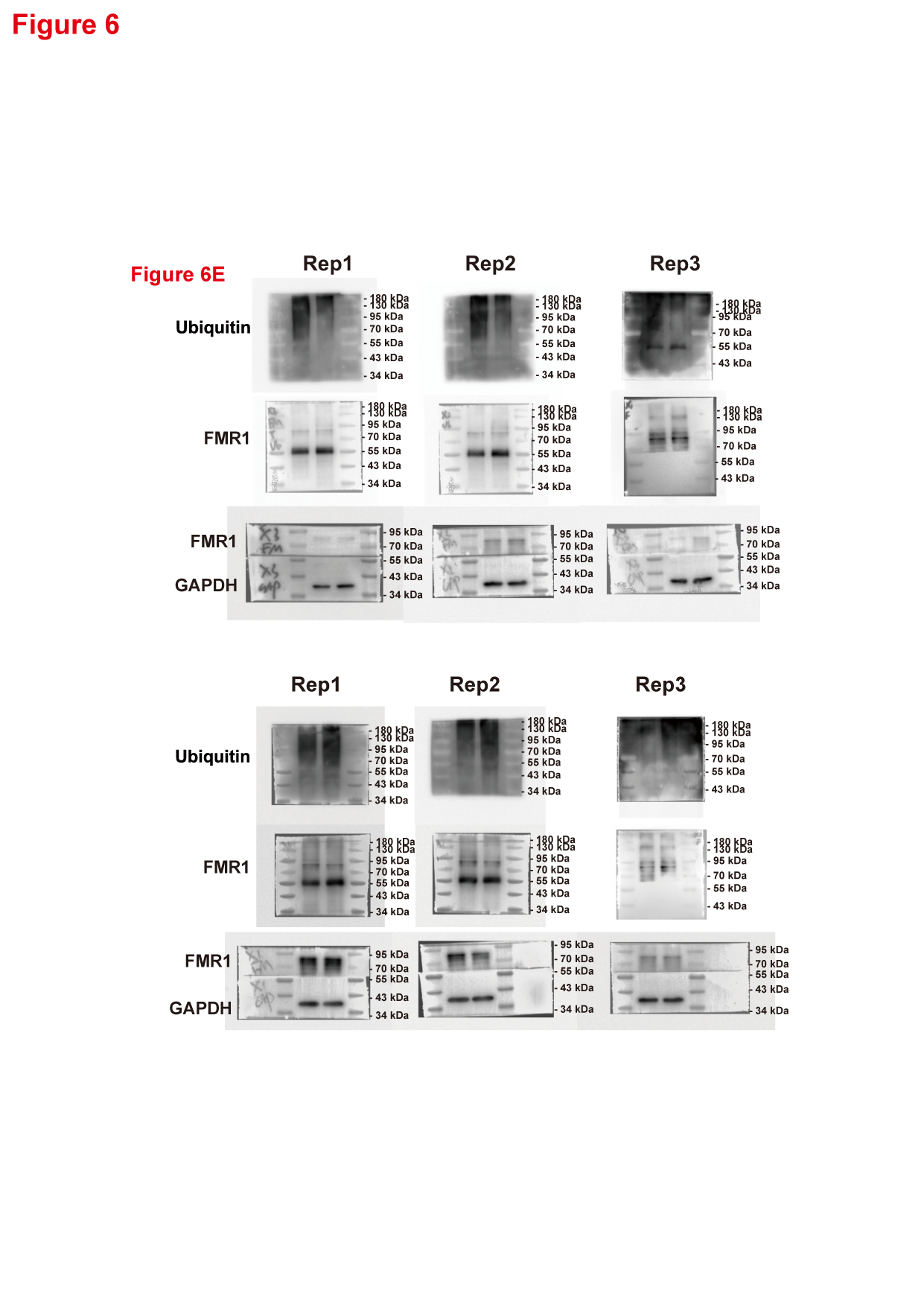

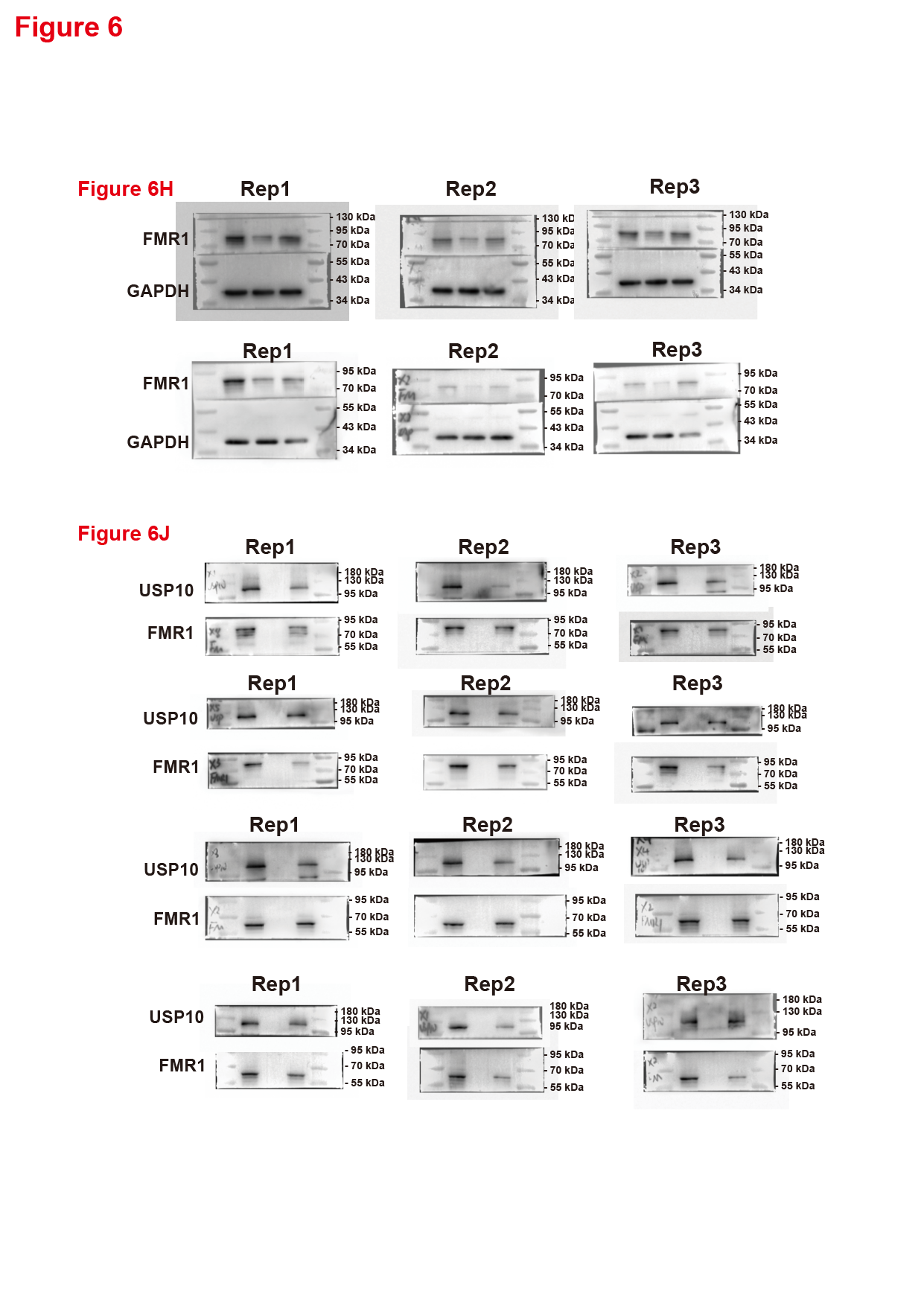


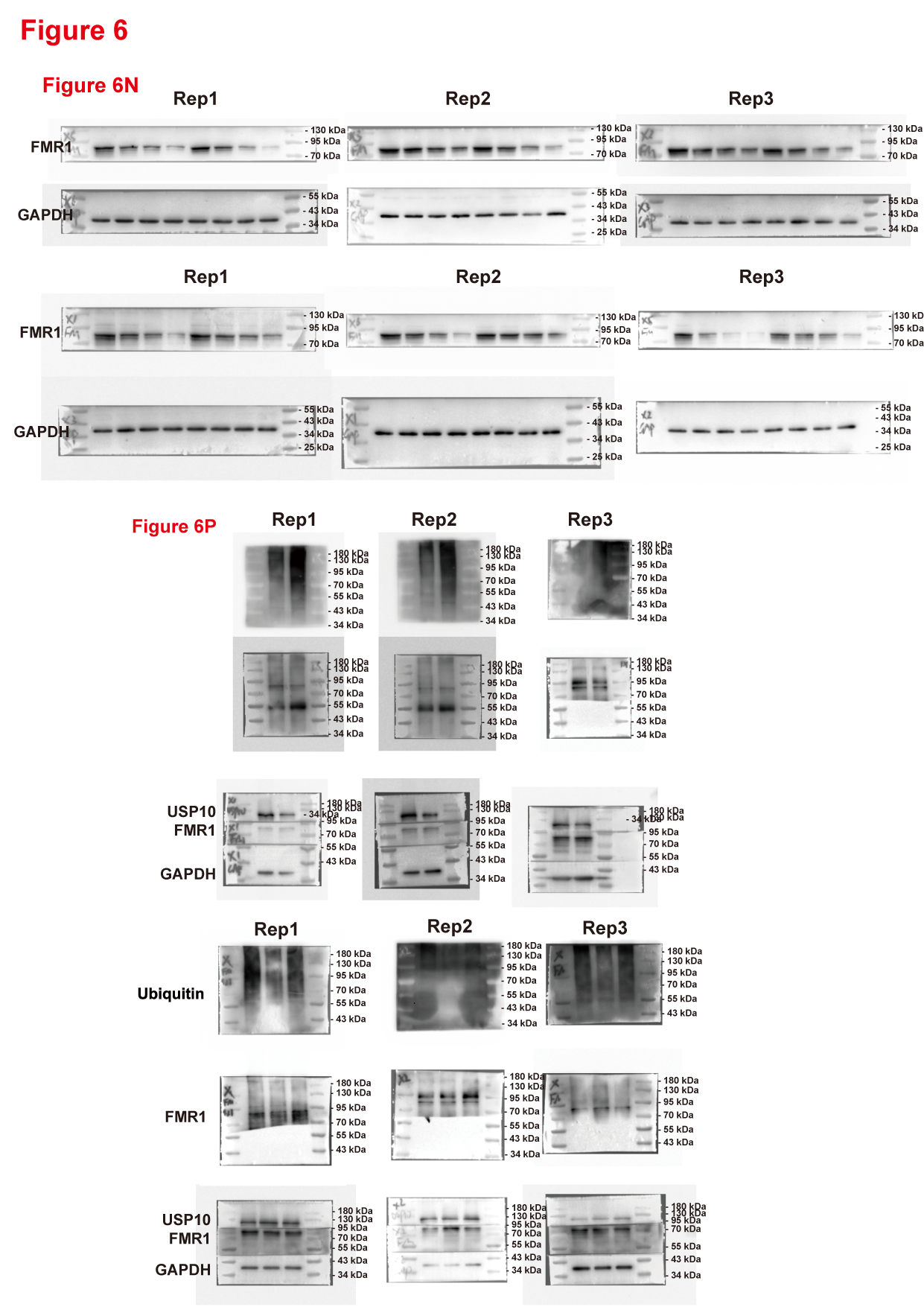


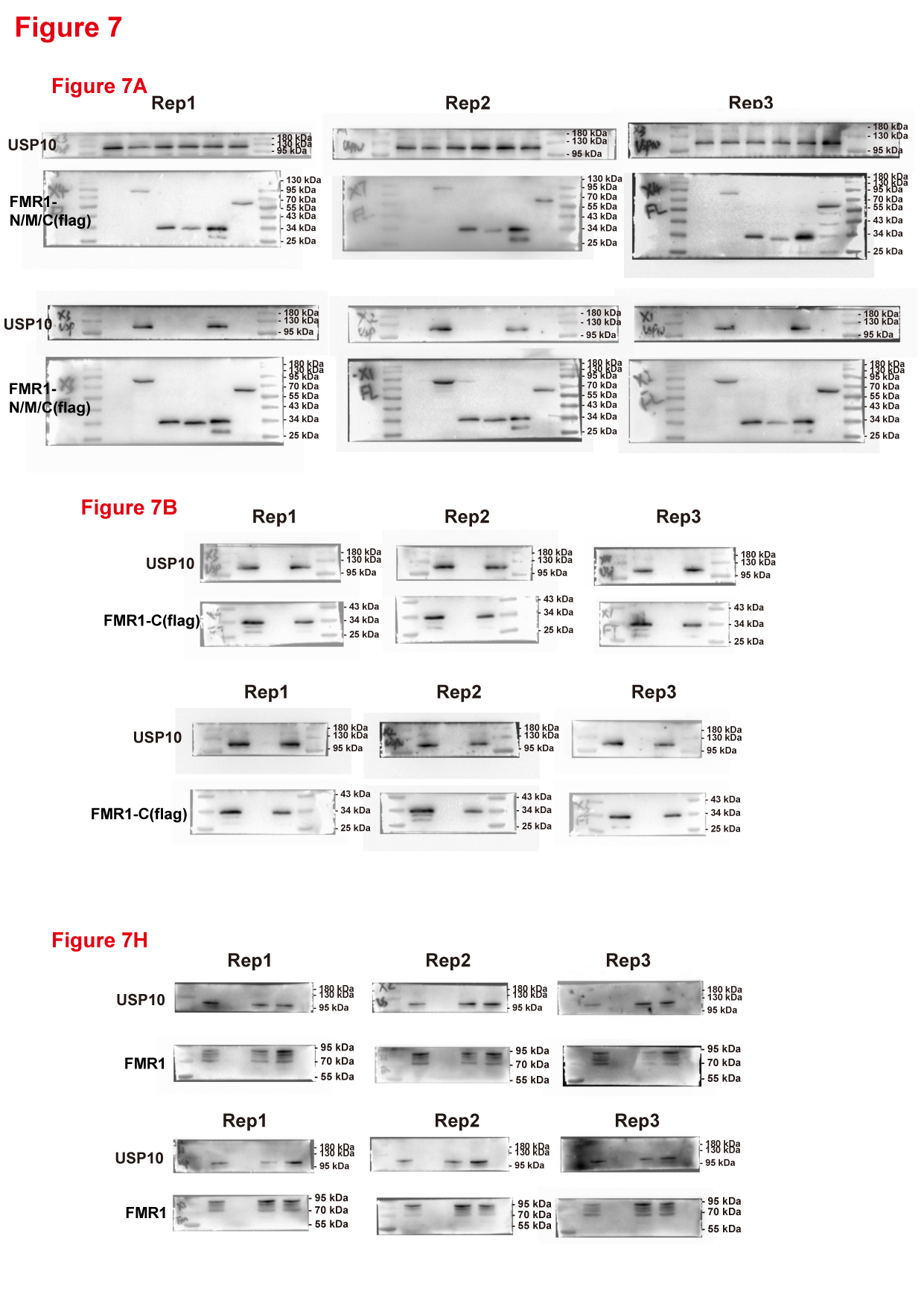


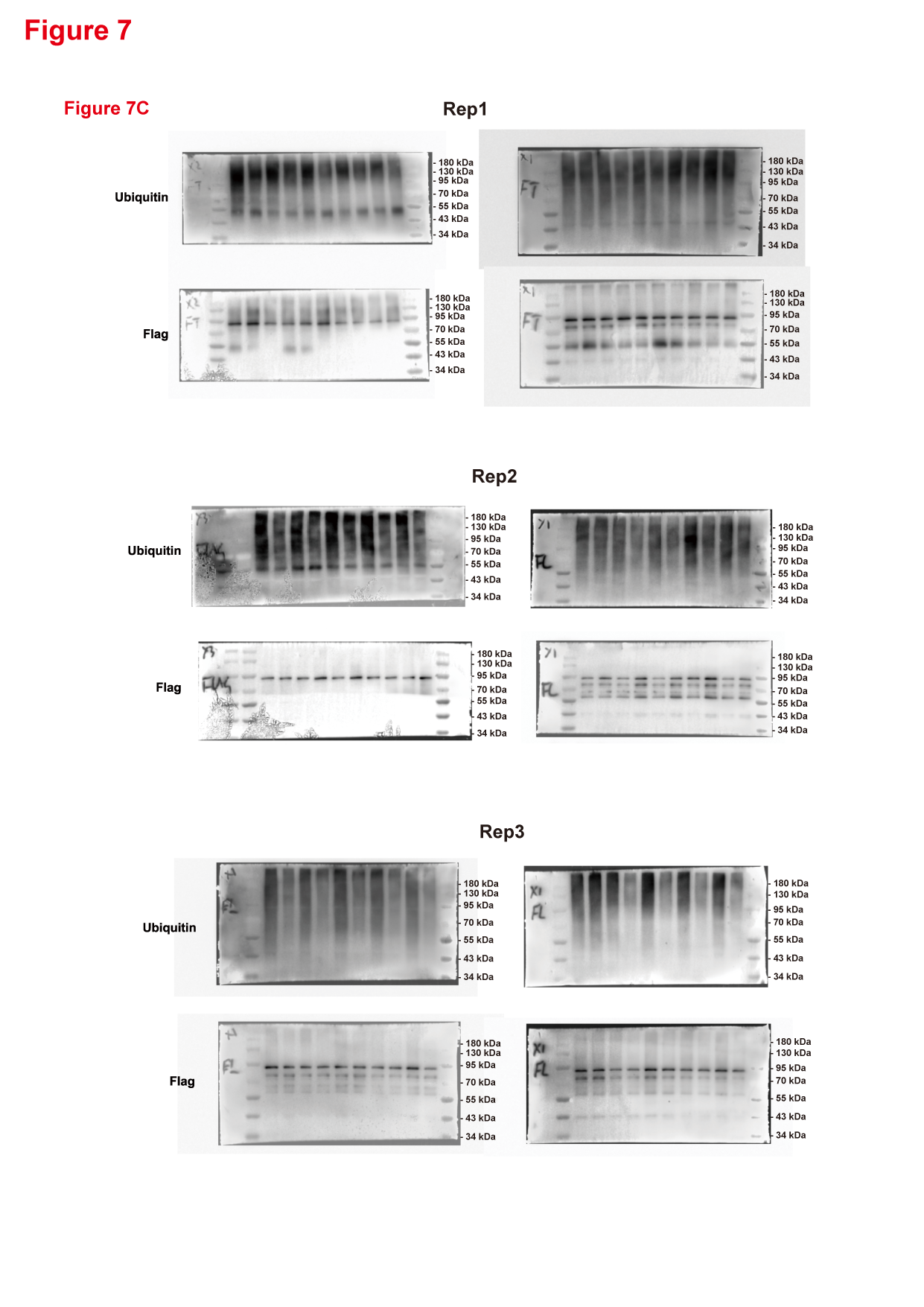

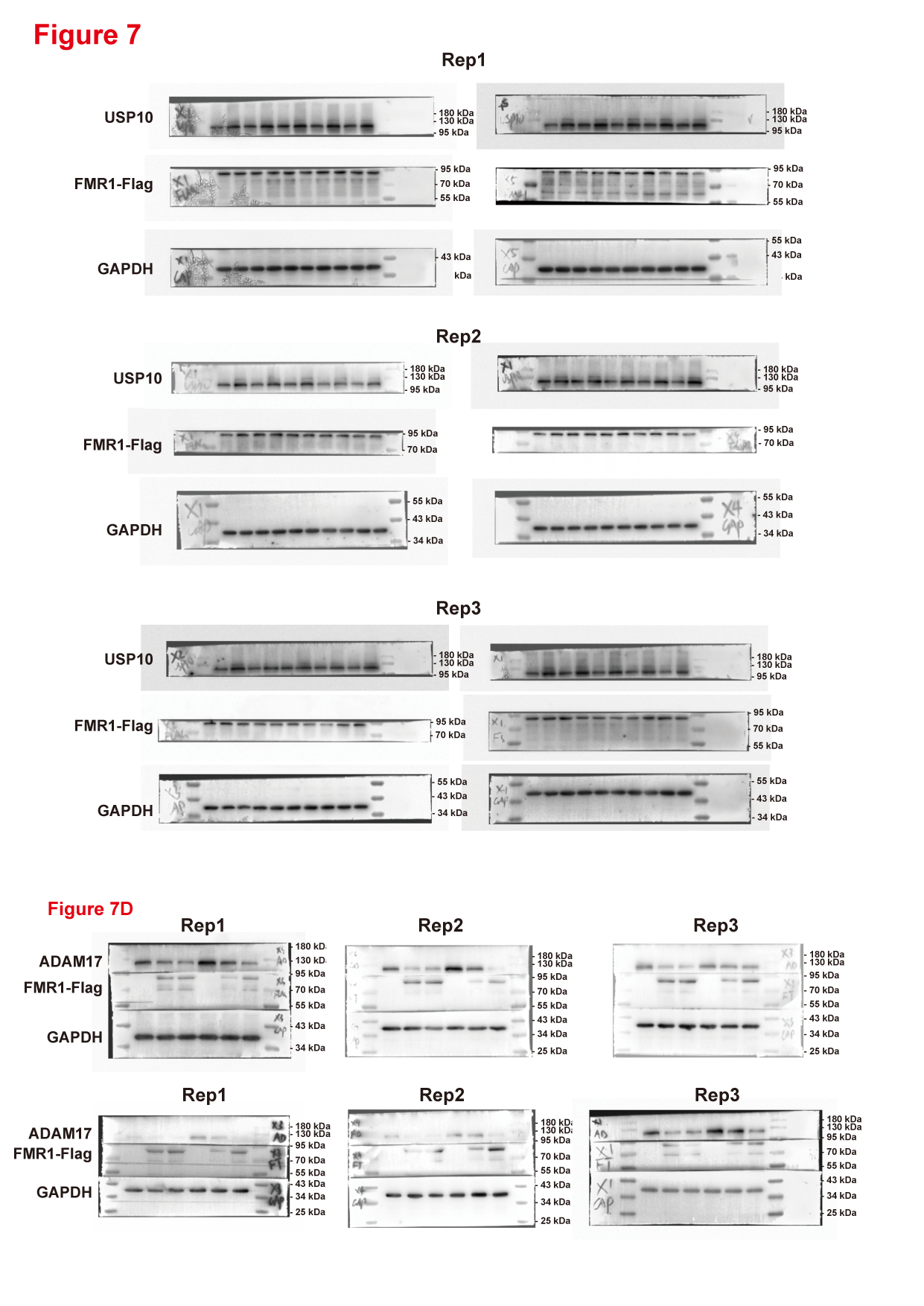

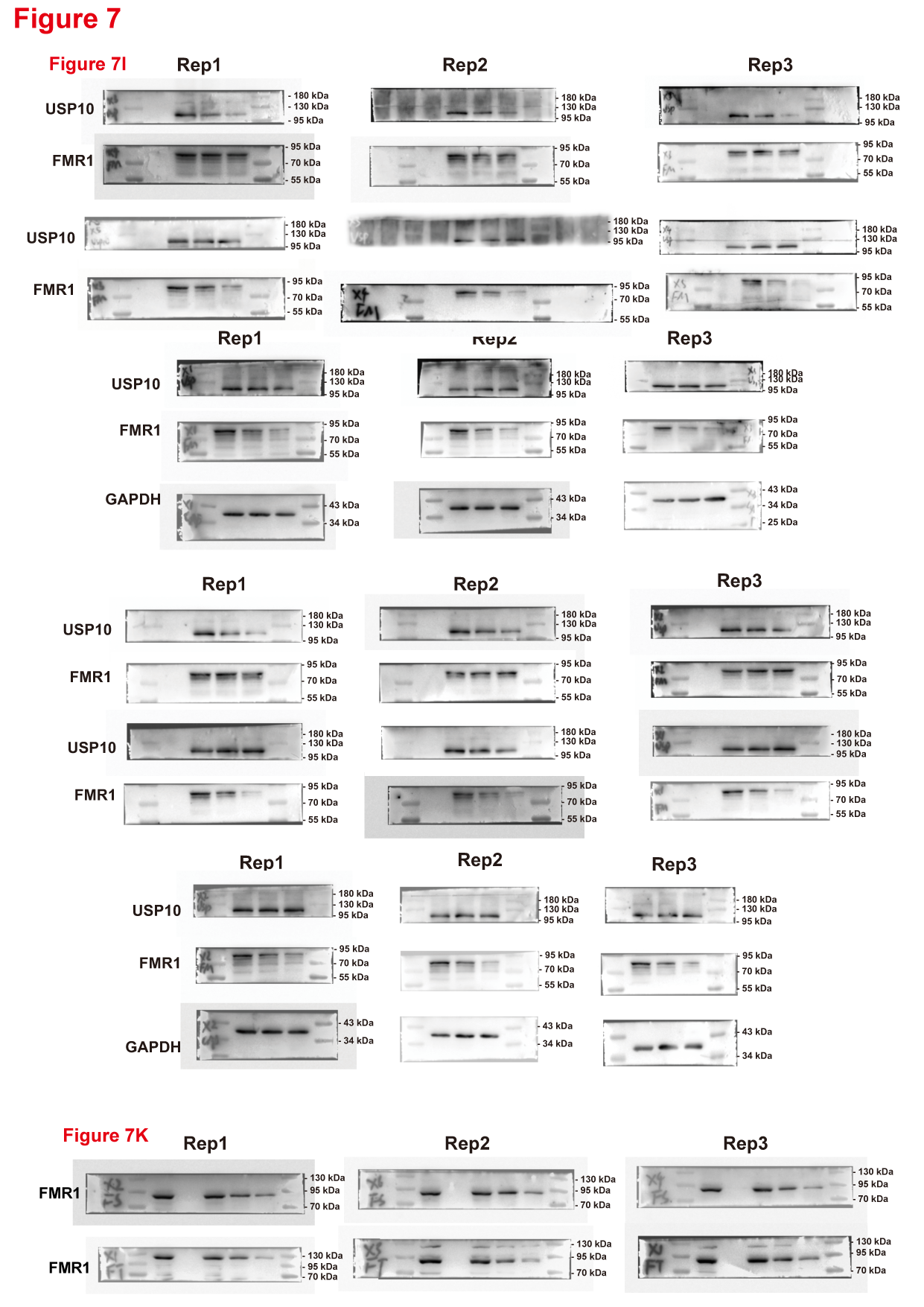


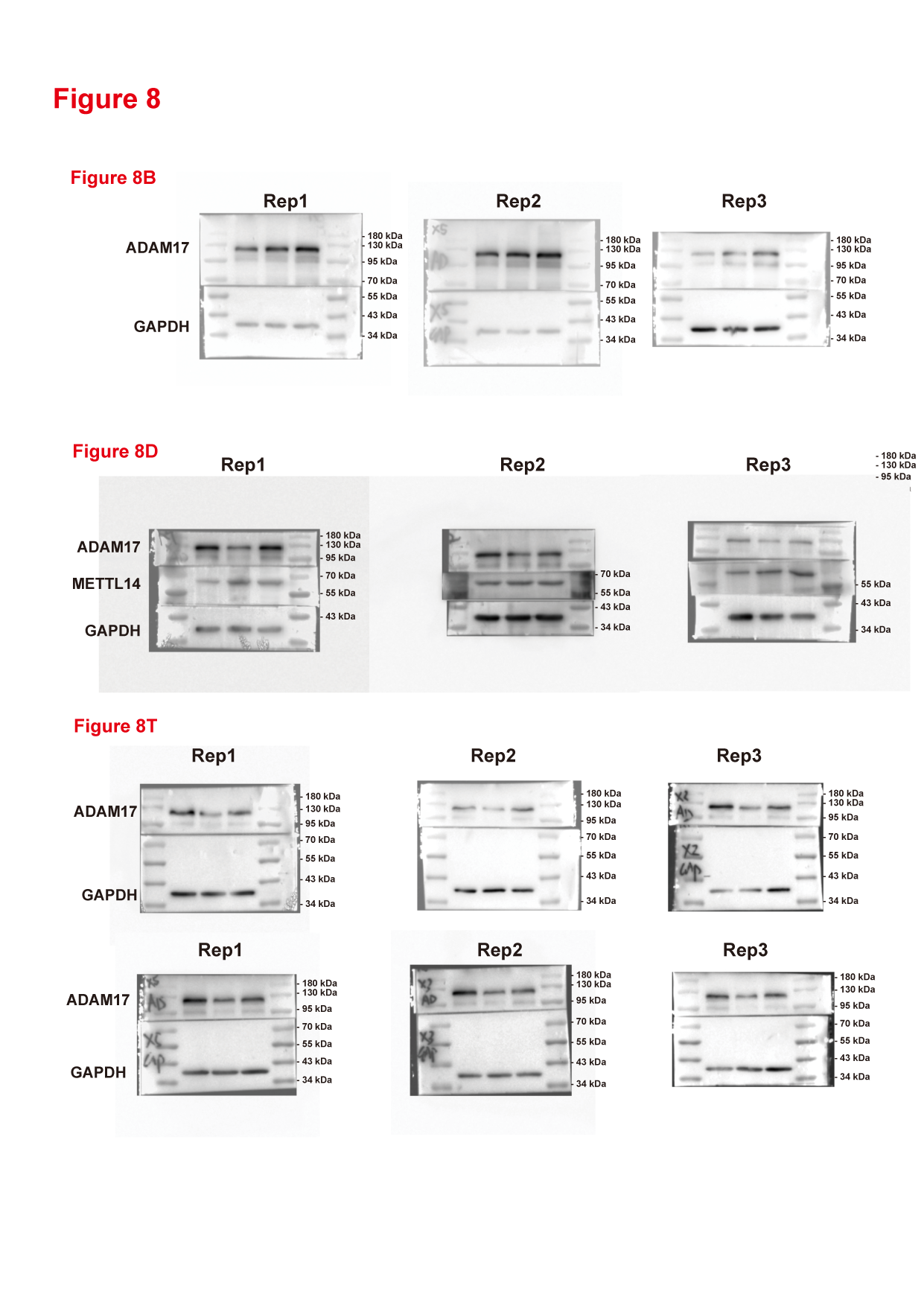

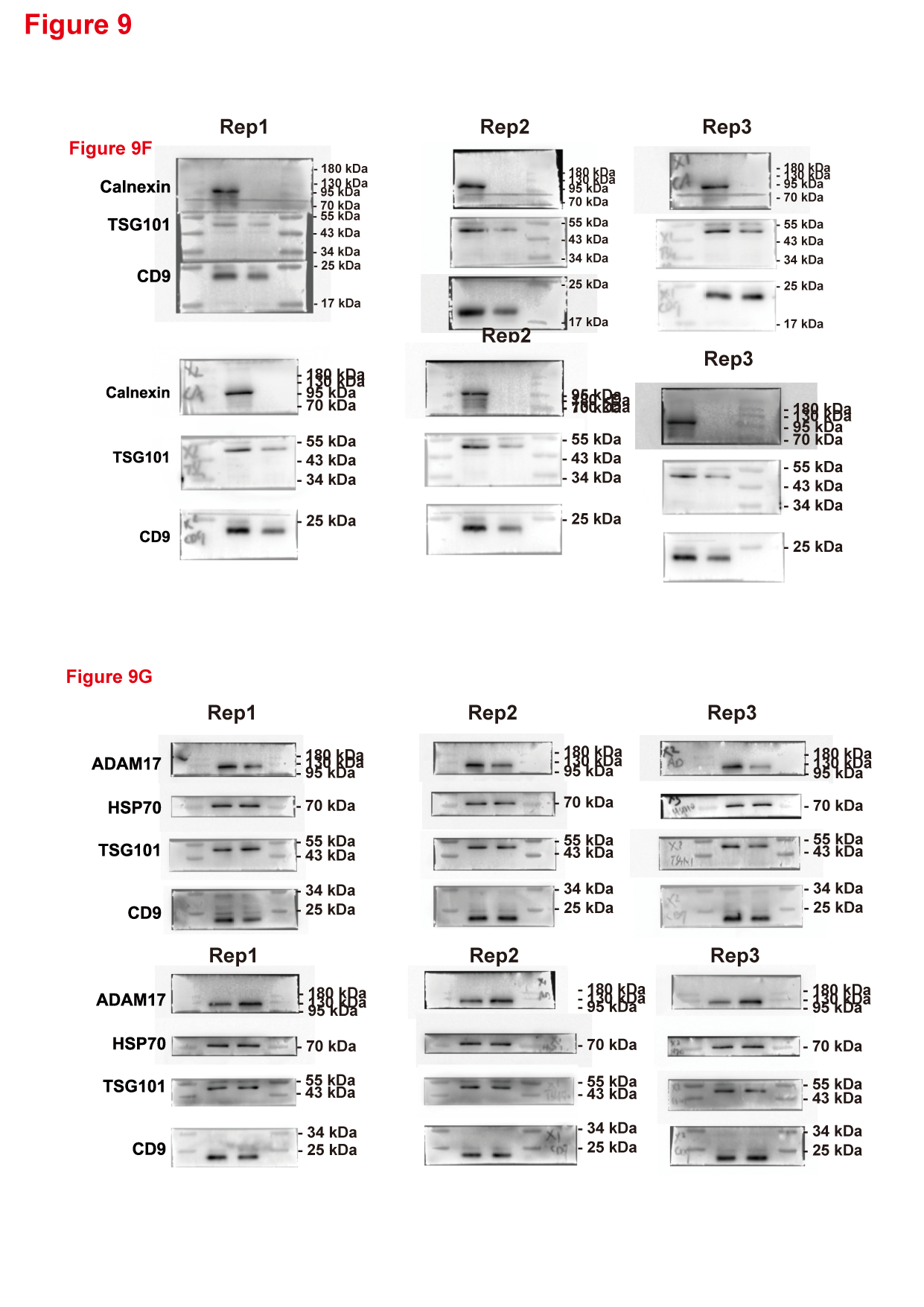

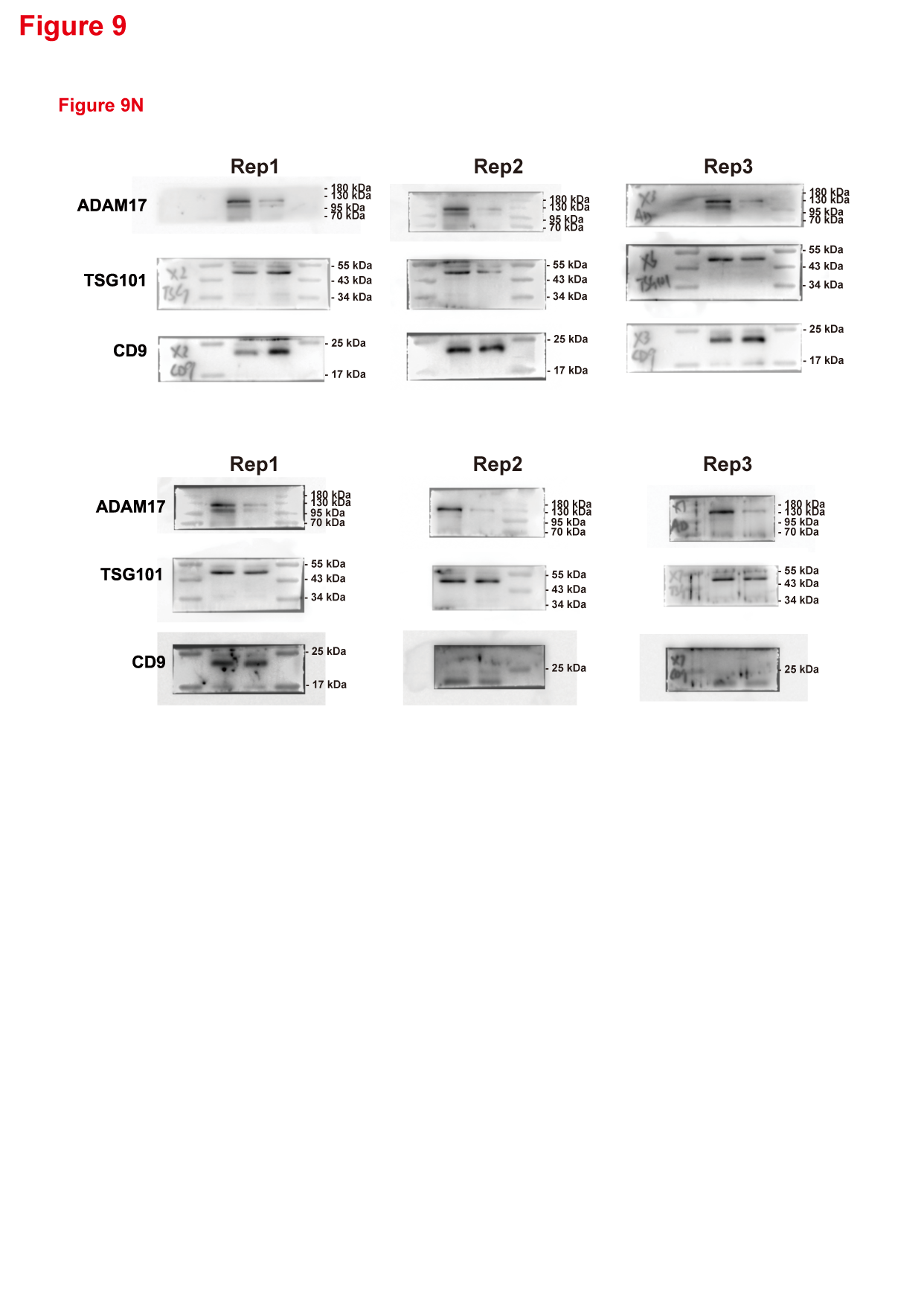

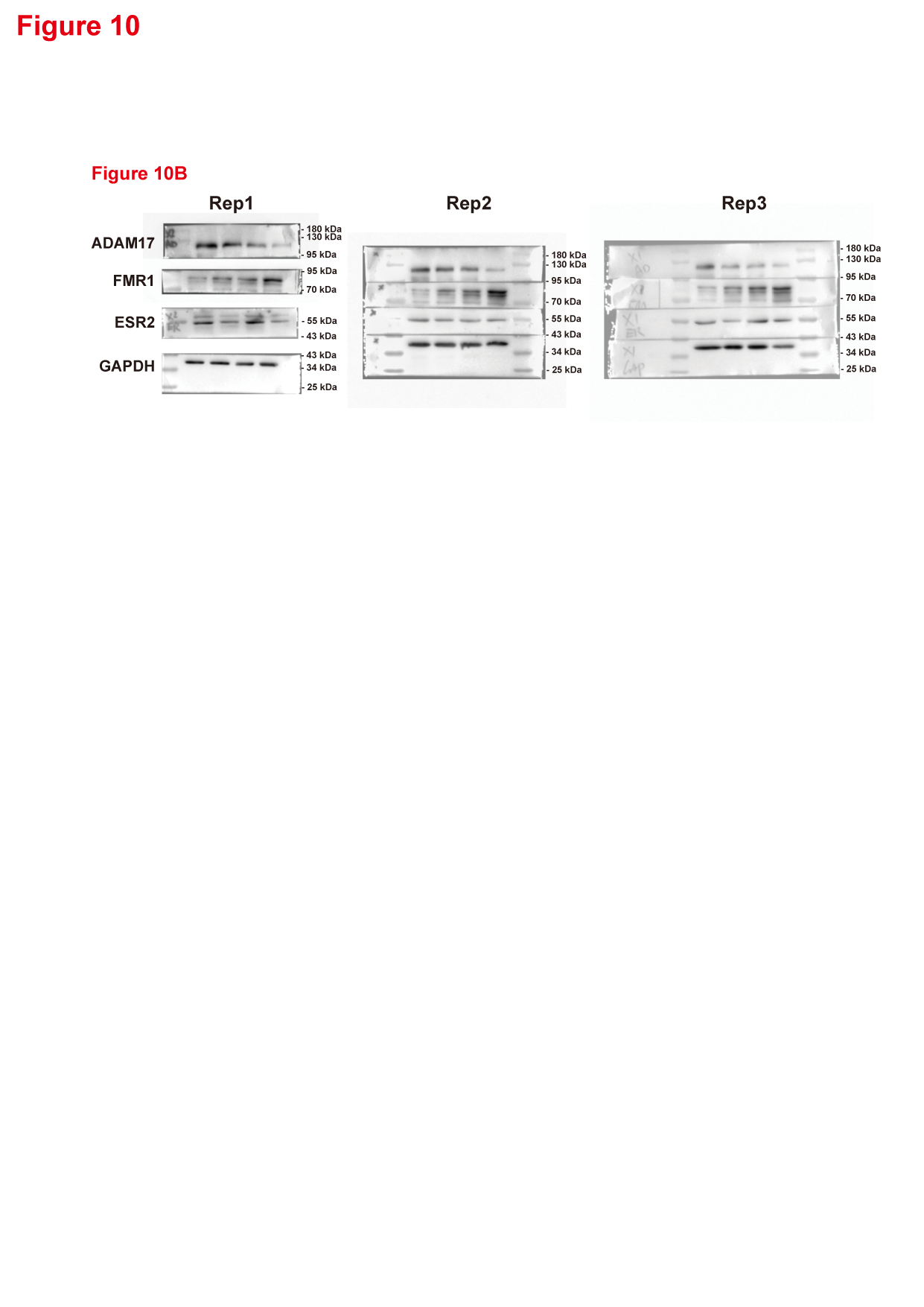


**Supplementary Figure 9.** **Uncropped western blot images from three independent replicates.The original, uncropped blots corresponding to the figures in the main text are shown.** The target proteins and their corresponding molecular weights are indicated on the blots. The numbers on the right side of each blot denote their respective positions in the main figures. Uncropped blots from three independent biological replicates are provided.

**Supplementary Table 1. The sequences of primers for qRT-PCR analysis**

| **Gene** | **Forward Primer** | **Reverse Primer** |
| --- | --- | --- |
| GAPDH  ESR2  hsa_circ_0001414  hsa_circ_0002699  hsa_circ_0001062  hsa_circ_0000317  hsa_circ_0015004  hsa_circ_0065217  hsa_circ_0020303  hsa_circ_0030431  hsa_circ_0001968  hsa_circ_0031594  AHNAK  U6  ERE#1  ERE#2  ADAM17  NOS3  P2RX4  PDPK1  STAT5A  TGFB1  TMSB4X  VEGFA  FMR1  FUS  USP10  USP11  METTL3  METTL14  MeRIP(327-451)  MeRIP(511-614)  MeRIP(2057-2159)  MeRIP(2475-2583)  MeRIP(2668-2868) | GGAGCGAGATCCCTCCAAAAT  TTCAAAGAGGGATGCTCACTTC  CAGAATGGGGTGATCGCTCT  ACTTCTTCAACAAGATCGTCAACA  TGAGAAAGCCTACAGAAAATCAA  GTGATCTGAAAGGGCCAGAG  GAGTGCAGGGGTCTCTCTTT  TGAGCCTCTACACCAGGAAGA  GCTCTATGCTGTGTACCTTCTGGA  CGGACTCGGAGAAGAACAGG  GGGATCTGATGTCTGGAGCAC  TCATAGCAGATGTGGAGCCC  TGCCTGAGATTGCTACTGGTG  GCTTCGGCAGCACATATACTAAAAT  GGTCCTCACCCCCGTATG  CCTTGGCCAGGTCTCCTTA  GACTCTAGGGTTCTAGCCCAC  TGATGGCGAAGCGAGTGAAG  CTACCAGGAAACTGACTCCGT  GGAACAGCGCAGTACGTTTCT  GCAGAGTCCGTGACAGAGG  GGCCAGATCCTGTCCAAGC  GACAGAGACGCAAGAGAAAAATC  AGGGCAGAATCATCACGAAGT  TATGCAGCATGTGATGCAACT  ATGGCCTCAAACGATTATACCCA  ATTGAGTTTGGTGTCGATGAAGT  CATTGAACGCAAGGTCATAGAGC  TTGTCTCCAACCTTCCGTAGT  GAACACAGAGCTTAAATCCCCA  GCTTGATTCTTTGCTCTCAGAC  GTCGTGGTGGTGGATGGTAA  GTGTGCCCTATGTCGATGCT  AGCTCCAAAACTGGACCACC  TGACAGCAAAGAAACAGAGTGC | GGCTGTTGTCATACTTCTCATGG  CCTTCACACGACCAGACTCC  TGATATGGCAGCGACAAATGC  TGGGAAGCATTCTGCTAAACTCT  TTTCTTCAAATCCTGCATCG  TCCACATCACCCTTCACCTT  CAACGTGTCAAGTGTCGGAT  TTGTGCCAGGGAAACACTGA  TCTCATTGATGGATGGATTGCC  AGGCGTCCTCATTATCTTTGCT  CAGAGCCACCATCATCAAACTG  TGTCCACGTGGCGAACATAA  CCTTTCAGTTTAGGAGACCCAAG  CGCTTCACGAATTTGCGTGTCAT  GCACAGAACTGGCTTGGATT  TCCACACTCCCTTTCTCTTCTC  GGAGACTGCAAACGTGAAACAT  ACTCATCCATACACAGGACCC  GGTATCACATAATCCGCCACAT  CTCGTTTCCAGCTCGGAATGG  CCACAGGTAGGGACAGAGTCT  GTGGGTTTCCACCATTAGCAC  CGCCAATATGCACTGTACATTCC  AGGGTCTCGATTGGATGGCA  TTGTGGCAGGTTTGTTGGGAT  GTAACTCTGCTGTCCGTAGGG  GGAGCCATAGCTTGCTTCTTTAG  AACAGTGTGAGATTTGCCCAA  CCAGATCAGAGAGGTGGTGTAG  TGTCAGCTAAACCTACATCCCTG  TCAAAGCTGAAAAAGTTAGTAGTGT  TGGGCTAGAACCCTAGAGTCA  ACTCGTTTCTCACATTTGCCAT  GCTGCTATTTGGGAAGGGGT  CCTGCAGGAAGTTCAAACACA |
